# Supplementary material for: ISO, via Upregulating MiR-137 Transcription, Inhibits GSK3β-HSP70-MMP-2 Axis, Resulting in Attenuating Urothelial Cancer Invasion
Source: Mol Ther Nucleic Acids. 2018 Jul 4;12:337–49. doi: 10.1016/j.omtn.2018.05.017 (PMC6037888; doi:10.1016/j.omtn.2018.05.017)
Supplement: Document S1. Figures S1–S4 [file mmc1.pdf]

OMTN, Volume *12*

Supplemental Information

ISO, via Upregulating MiR-137 Transcription, Inhibits GSK3β-HSP70-MMP-2 Axis, Resulting in Attenuating Urothelial Cancer Invasion

Xirui Guo, Haishan Huang, Honglei Jin, Jiheng Xu, Sanjiv Risal, Jingxia Li, Xin Li, Huiying Yan, Xingruo Zeng, Lei Xue, Changyan Chen, and Chuanshu Huang

Supplement Fig. 1

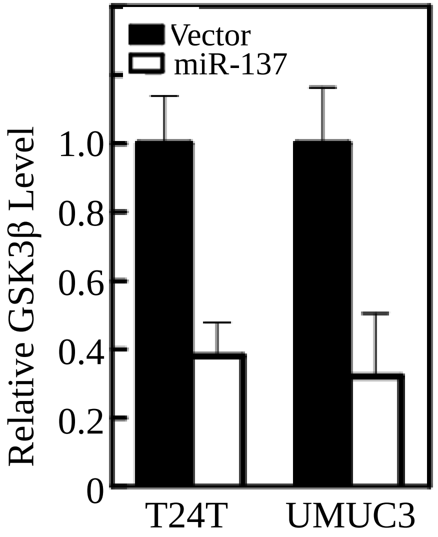

SFig. 1A, Quantitative results obtained from triplicated experiments shown in Fig. 3A.

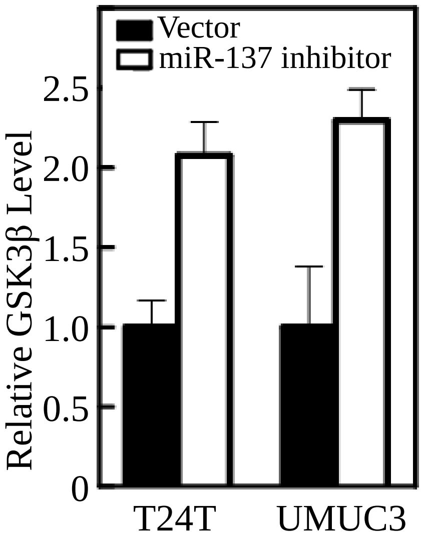

SFig. 1B, Quantitative results obtained from triplicated experiments shown in Fig. 3B.

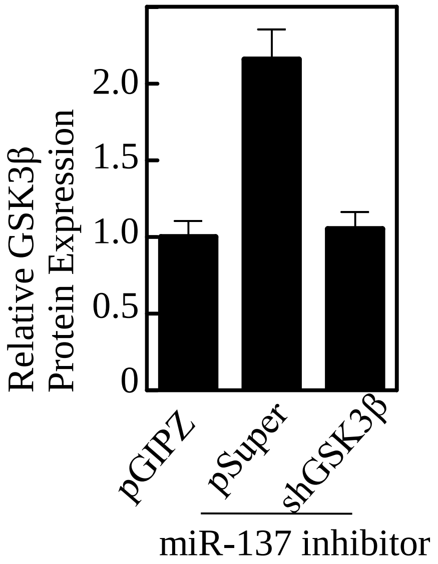

SFig. 1C, Quantitative results obtained from triplicated experiments shown in Fig. 3C.

Supplement Fig. 2

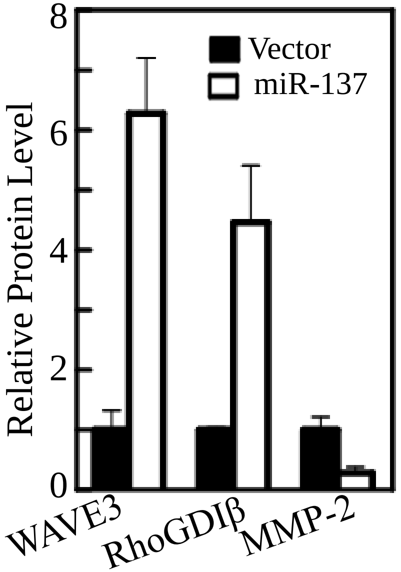

SFig. 2A, Quantitative results obtained from triplicated experiments shown in Fig. 5A.

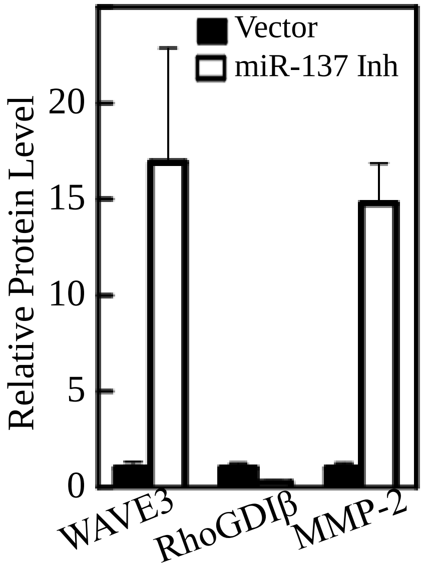

SFig. 2B, Quantitative results obtained from triplicated experiments shown in Fig. 5B.

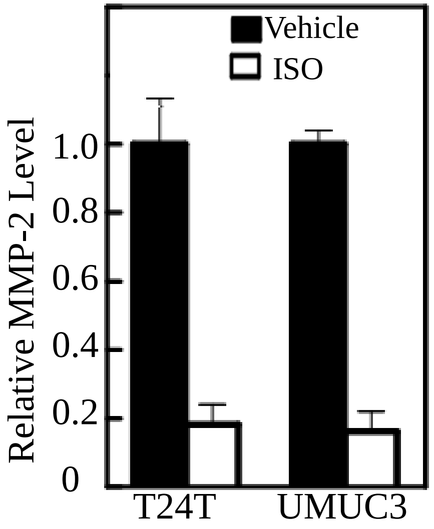

SFig. 2C, Quantitative results obtained from triplicated experiments shown in Fig. 5C.

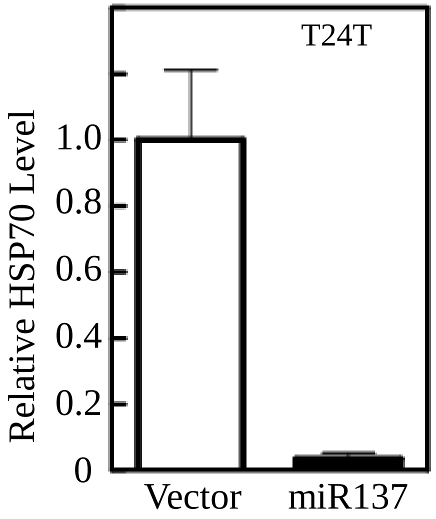

SFig. 2D, Quantitative results obtained from triplicated experiments shown in Fig. 5E.

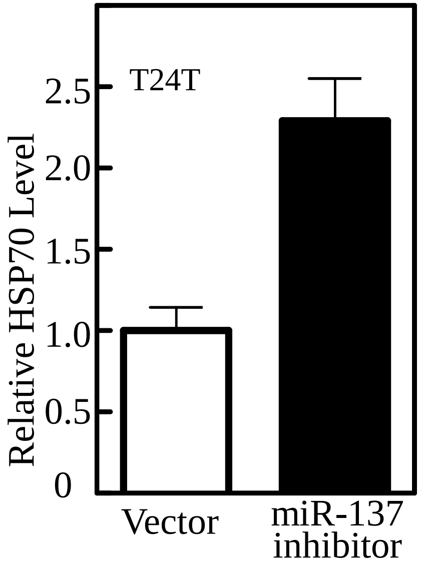

SFig. 2E, Quantitative results obtained from triplicated experiments shown in Fig. 5F.

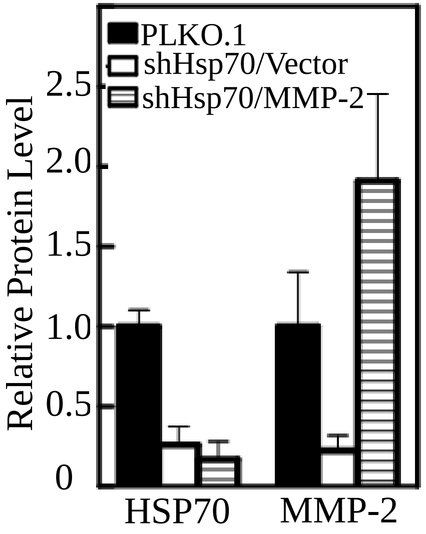

SFig. 2F, Quantitative results obtained from triplicated experiments shown in Fig. 5G.

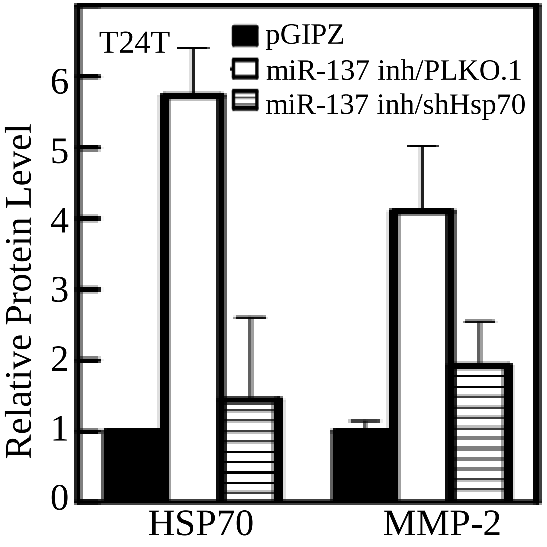

SFig. 2G, Quantitative results obtained from triplicated experiments shown in Fig. 5J.

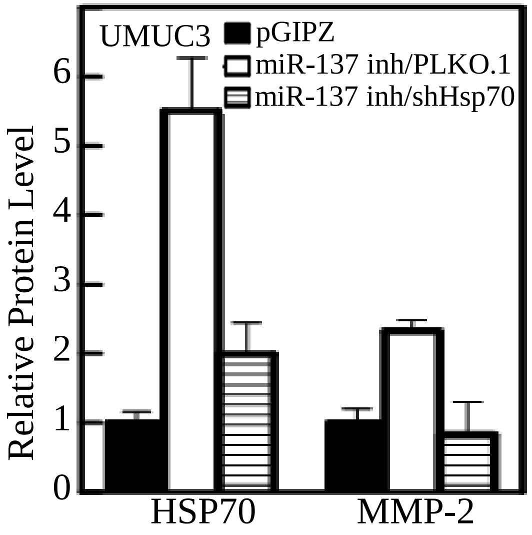

SFig. 2H, Quantitative results obtained from triplicated experiments shown in Fig. 5K.

Supplement Fig. 3

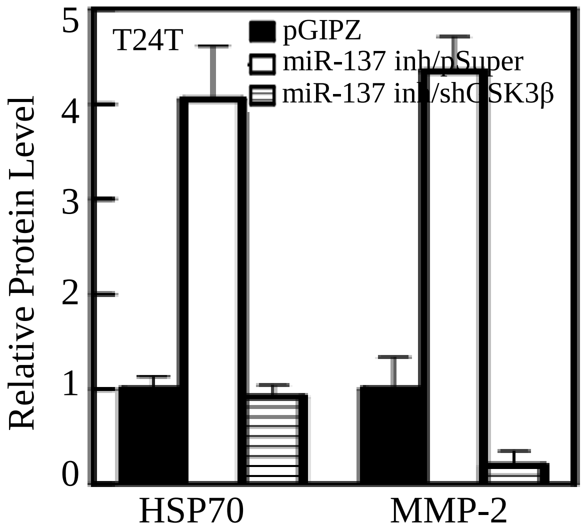

SFig. 3A, Quantitative results obtained from triplicated experiments shown in Fig. 6A.

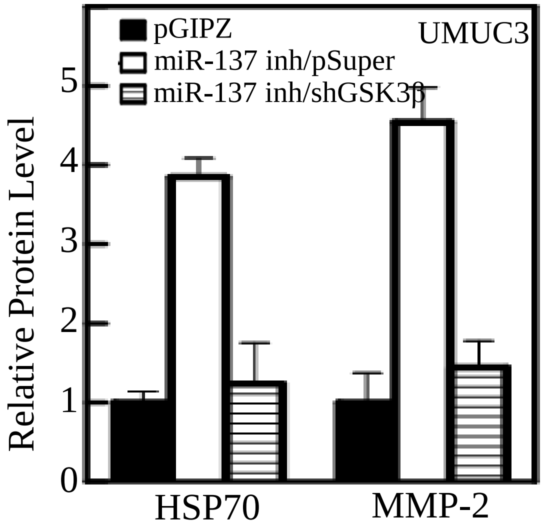

SFig. 3B, Quantitative results obtained from triplicated experiments shown in Fig. 6B.

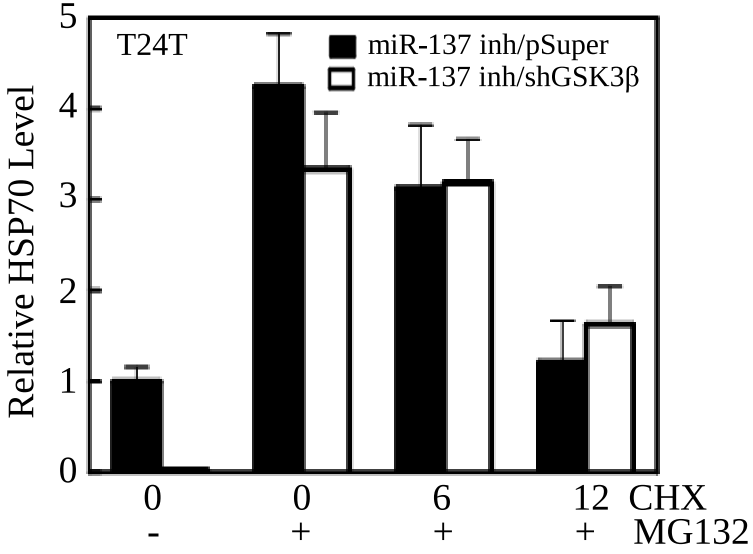

SFig. 3C, Quantitative results obtained from triplicated experiments shown in Fig. 6D.

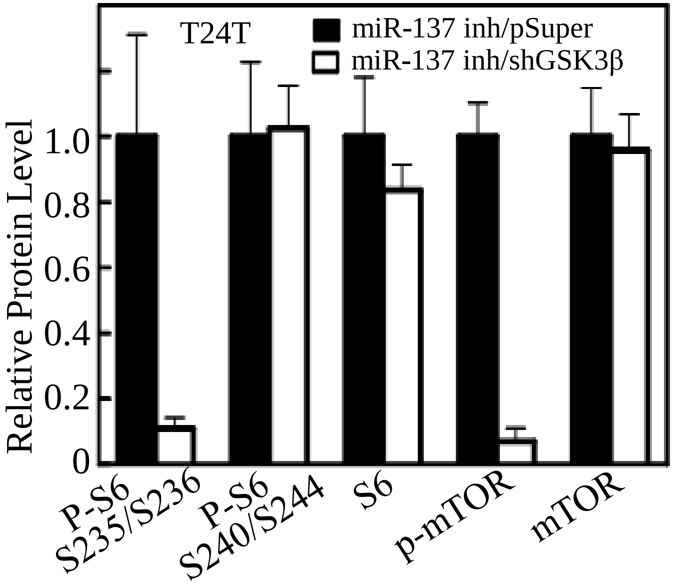

SFig. 3D, Quantitative results obtained from triplicated experiments shown in Fig. 6E.

Supplement Fig. 4

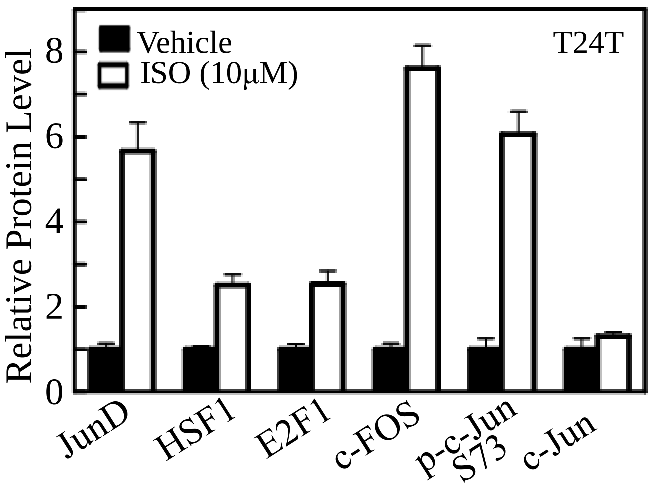

SFig. 4A, Quantitative results obtained from triplicated experiments shown in Fig. 7D.

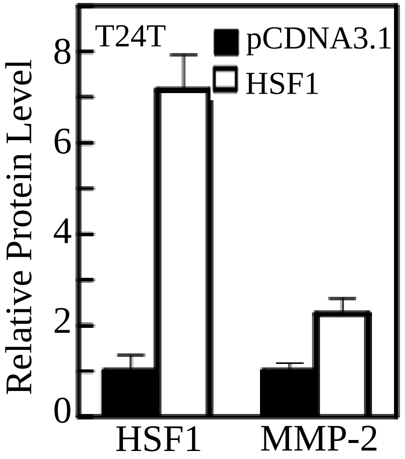

SFig. 4C, Quantitative results obtained from triplicated experiments shown in Fig. 7F.

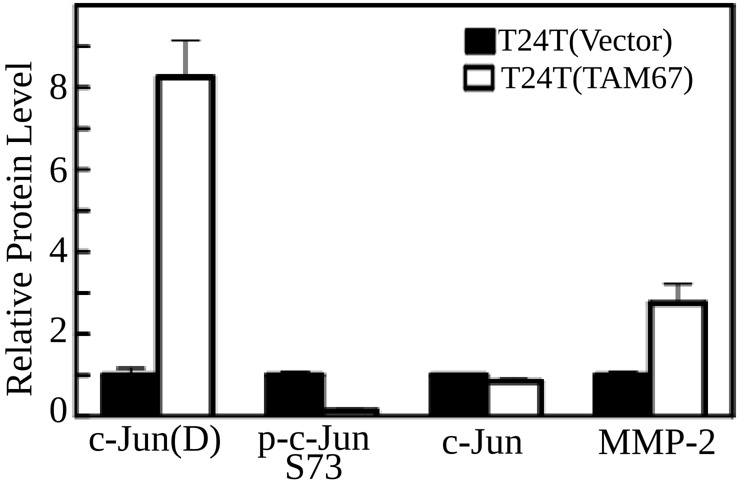

SFig. 4E, Quantitative results obtained from triplicated experiments shown in Fig. 7J.

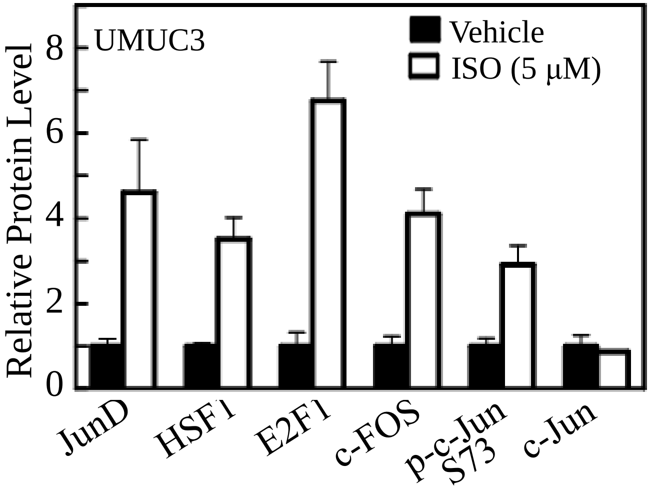

SFig. 4B, Quantitative results obtained from triplicated experiments shown in Fig. 7E.

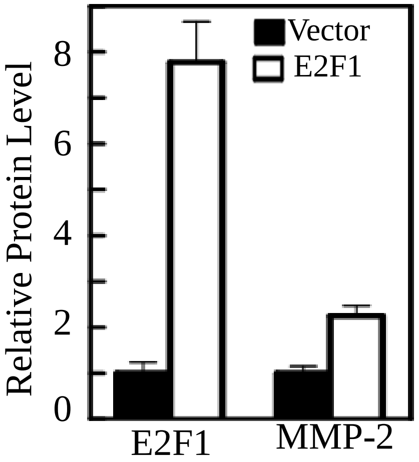

SFig. 4D, Quantitative results obtained from triplicated experiments shown in Fig. 7H.

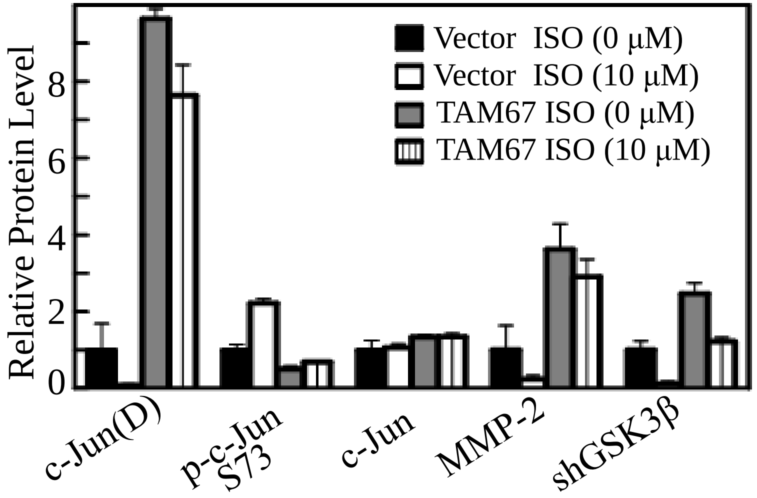

SFig. 4F, Quantitative results obtained from triplicated experiments shown in Fig. 7M.
