# Supplementary material for: ISO, via Upregulating MiR-137 Transcription, Inhibits GSK3β-HSP70-MMP-2 Axis, Resulting in Attenuating Urothelial Cancer Invasion
Source: Mol Ther Nucleic Acids. 2018 Jul 4;12:337–49. doi: 10.1016/j.omtn.2018.05.017 (PMC6037888; doi:10.1016/j.omtn.2018.05.017)
Supplement: Document S2. Article plus Supplemental Information [file mmc2.pdf]

Molecular Therapy
Nucleic Acids
Original Article

AMERICAN SOCIETY of GENE & CELL THERAPY

ISO, via Upregulating MiR-137 Transcription, Inhibits GSK3β-HSP70-MMP-2 Axis, Resulting in Attenuating Urothelial Cancer Invasion

Xirui Guo,[1,2,4] Haishan Huang,[2,4] Honglei Jin,[1,2,4] Jiheng Xu,[1,2] Sanjiv Risal,[3] Jingxia Li,[1] Xin Li,[2] Huiying Yan,[2] Xingruo Zeng,[1] Lei Xue,[1] Changyan Chen,[3] and Chuanshu Huang[1]

[1]Nelson Institute of Environmental Medicine, New York University School of Medicine, Tuxedo, NY 10987, USA; [2]School of Laboratory Medicine and Life Science, Wenzhou Medical University, Wenzhou, Zhejiang 325035, China; [3]The Center of Drug Discovery, Northeastern University, Boston, MA 02115, USA

Our most recent studies demonstrate that miR-137 is downregulated in human bladder cancer (BC) tissues, while treatment of human BC cells with isorhapontigenin (ISO) elevates miR-137 abundance. Since ISO showed a strong inhibition of invasive BC formation in the N-butyl-N-(4-hydroxybutyl) nitrosamine (BBN)-induced invasive BC mouse model, the elucidation of a potential biological effect of miR-137 on antagonizing BC invasion and molecular mechanisms underlying ISO upregulation of miR-137 are very important. Here we discovered that ectopic expression of miR-137 led to specific inhibition of BC invasion in human high-grade BC T24T and UMUC3 cells, while miR-137 deletion promoted the invasion of both cells, indicating the inhibitory effect of miR-137 on human BC invasion. Mechanistic studies revealed that ISO treatment induced miR-137 transcription by promoting c-Jun phosphorylation and, in turn, abolishing matrix metalloproteinase-2 (MMP-2) abundance and invasion in BC cells. Moreover, miR-137 was able to directly bind to the 3′ UTR of Glycogen synthase kinase-3β (GSK3β) mRNA and inhibit GSK3β protein translation, consequently leading to a reduction of heat shock protein-70 (HSP70) translation via targeting the mTOR/S6 axis. Collectively, our studies discover an unknown function of miR-137, directly targeting the 3′ UTR of GSK3β mRNA and, thereby, inhibiting GSK3β protein translation, mTOR/S6 activation, and HSP70 protein translation and, consequently, attenuating HSP70-mediated MMP-2 expression and invasion in human BC cells. These novel discoveries provide a deep insight into understanding the biomedical significance of miR-137 downregulation in invasive human BCs and the anti-cancer effect of ISO treatment on mouse invasive BC formation.

INTRODUCTION

Bladder cancer (BC) is the second urological malignancy and causes about 150,000 deaths annually worldwide.[1,2] Although during initial treatment, approximately 70% of non-muscle-invasive tumors in BC cases still recur, and only 20%–30% of them become to invade the lamina propria and are staged as T1.[3] Eventually, 10%–15% of the recurrent tumors progress to being muscle-invasive and metastasized tumors.[4,5] Since muscle-invasive urothelial carcinomas are responsible for almost 100% of death from this disease,[6] the identification of new molecular targets that specifically regulate the pathological process of BC invasion are of extreme importance for improving the clinical outcome of patients with this disease.

Isorhapontigenin (ISO) is a new natural derivative of stilbene and isolated from a Chinese herb *Gnetum cleistostachyum*; has been discovered to suppress several tumorigenic processes, including invasion,[7] proliferation,[8,9] apoptosis,[10] and autophagy;[11] and is a potential anti-cancer drug as a therapeutic regimen for BC patients. Our previous studies have demonstrated that ISO suppresses N-butyl-N-(4-hydroxybutyl) nitrosamine (BBN)-induced mouse invasive BC formation *in vivo* and human BC invasion *in vitro*.[7] Our studies also revealed that ISO treatment elevates miR-137 levels in T24T and UMUC3 cells.[12] In the light of these findings, we here investigated the upstream regulator for ISO upregulation of miR-137 and its potential effect on BC invasion in human BC cells.

MicroRNAs (miRNAs), known as a family of endogenous small non-coding RNAs 19–25 nt in length, negatively regulate target gene expression by base pairing with targeting mRNAs at 3′ UTRs, leading to mRNA degradation or translational repression.[13] Located on chromosome 1p22,[14] miR-137 has attracted much attention because it is frequently downregulated in cancer tissues, such as ovarian cancer,[15] gastric cancer,[16] colorectal cancer,[17] non-small-cell lung cancer,[18] and neuroblastoma.[19] It has also been reported that miR-137 can suppress invasion in thyroid cancer,[20] non-small-cell lung cancer,[18] and breast cancer.[21] Our previous studies indicated that miR-137 expression in BC tissues is almost undetectable in comparison to their adjacent non-tumorous bladder urothelium.[12] Although able to induce the

Received 27 September 2017; accepted 20 May 2018;
https://doi.org/10.1016/j.omtn.2018.05.017.

[4]These authors contributed equally to this work.

Correspondence: Chuanshu Huang, Nelson Institute of Environmental Medicine, New York University School of Medicine, 57 Old Forge Road, Tuxedo, NY 10987, USA.
E-mail: chuanshu.huang@nyumc.org

This is an open access article under the CC BY license (http://creativecommons.org/licenses/by/4.0/).

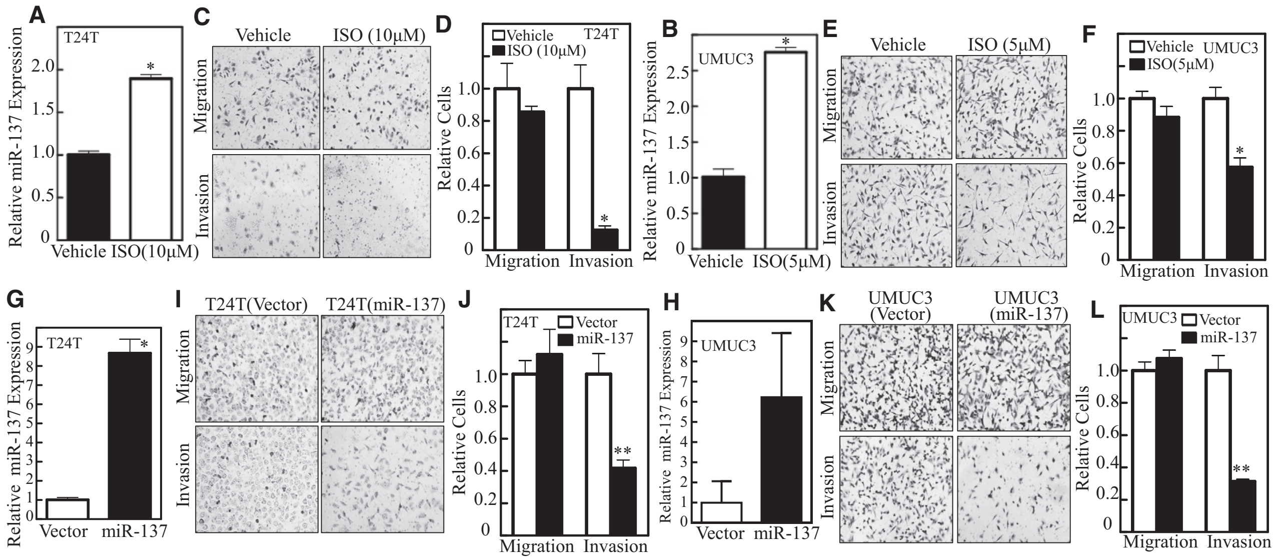

Figure 1. ISO and miR-137 Specifically Suppressed the Invasion Ability of Bladder Cancer in Both T24T and UMUC3 Cells

(A–F) The relative expression levels of miR-137 were evaluated by real-time qPCR in T24T (A) and UMUC3 (B) cells, followed by ISO (10 and 5 μM) treatment for 12 hr. Human T24T (C and D) and UMUC3 (E and F) BC cells were cultured in chamber or pre-coated matrigel chamber and treated with medium containing either vehicle or the indicated concentration of ISO for 24 hr. The cells were then fixed and stained. The migration and invasion rates of T24T (D) and UMUC3 (F) were quantified by counting the relative migrated (transwell) and invaded cells in at least three random fields under a light microscope. (G–L) Confirmation of miR-137 overexpression by qRT-PCR in T24T (G) and UMUC3 (H) cells stably transfected with pcDNA3.2/V5-mmu-miR-137. Invasion abilities of miR-137 overexpression in T24T (I and J) and UMUC3 (K and L) cells were determined using BD BioCoat Matrigel Invasion Chamber. The migration ability was determined by using the empty insert membrane without the matrigel. The invasion ability of miR-137 overexpression in T24T (J) and UMUC3 (L) cells was assessed by using the same system except that the matrigel was applied. The invasion rate was normalized with the insert control, according to the manufacturer's instruction, and the values shown are mean ± SD (n = 3). *p < 0.05; **p < 0.01.

expression of miR-137 by ISO treatment in human BC cells,[12] the underlying mechanisms responsible for ISO upregulation of miR-137 and thereby suppression of BC invasion have never been explored. In this study, we investigated how ISO affected miR-137 abundance and how ISO-initiated miR-137 upregulation contributed to the inhibition of BC invasion.

RESULTS

miR-137 Induction Was Crucial for ISO Inhibition of Invasion in Human BC Cells

Our previous studies have demonstrated that ISO treatment increases miR-137 expression[12] and suppressed invasion in human BC cells.[7] Our published work also indicated that IC_{50} for T24T in monolayer culture is 55.2 μM, while UMUC3 is more sensitive to ISO treatment.[10] To explore the potential effect and mechanisms of ISO on BC invasion, non-toxic doses of ISO, including 10 μM for T24T cells and 5 μM for UMUC3 cells, were selected in the current experiments, which we also employed in our most recent study.[22] As the continuation of our investigation, T24T and UMUC3 cells were treated with ISO for 12 hr, and the expression of miR-137 was evaluated. Consistent with our previous findings, the expression of this microRNA was upregulated in both cells (Figures 1A and 1B). In addition, the relative invasion activities, but not migration, were specifically abolished in UMUC3 and T24T cells by the addition of ISO to the experimental system (Figures 1C–1F).

To test the potential contribution of miR-137 to ISO inhibition of BC invasion, we stably transfected the miR-137 expression construct into T24T and UMUC3 cells, and the effect of miR-137 on BC invasion was evaluated. As shown in Figures 1G and 1H, ectopic expression of miR-137 was observed in both transfectants by qRT-PCR. As expected, introduction of miR-137 resulted in a dramatic attenuation of invasion without significantly affecting migration abilities in either the T24T cells or the UMUC3 cells (Figures 1I–1L). Subsequently, T24T vector and T24T miR-137 inhibitor cells were employed to test whether increased miR-137 expression by ISO mediated its inhibition of BC invasion. The ectopic expression levels of miR-137 in T24T cells were analyzed. The introduction of miR-137 inhibitor into T24T cells abolished miR-137 induction by ISO and specifically reversed the ISO inhibition of BC invasion without affecting the migration (Figures 2A–2D). Consistent results were also observed in UMUC3 cells that were transfected with miR-137 inhibitor (Figures 2E–2G). Taken together, these data consistently demonstrated that ISO is a new natural compound that specifically inhibits human BC invasion by promoting miR-137 expression.

miR-137 Inhibited BC Cell Invasion through the Suppression of GSK3β

It was reported that GSK3β inhibition causes a significant dysregulation of actin cytoskeleton organization and insufficiency of focal

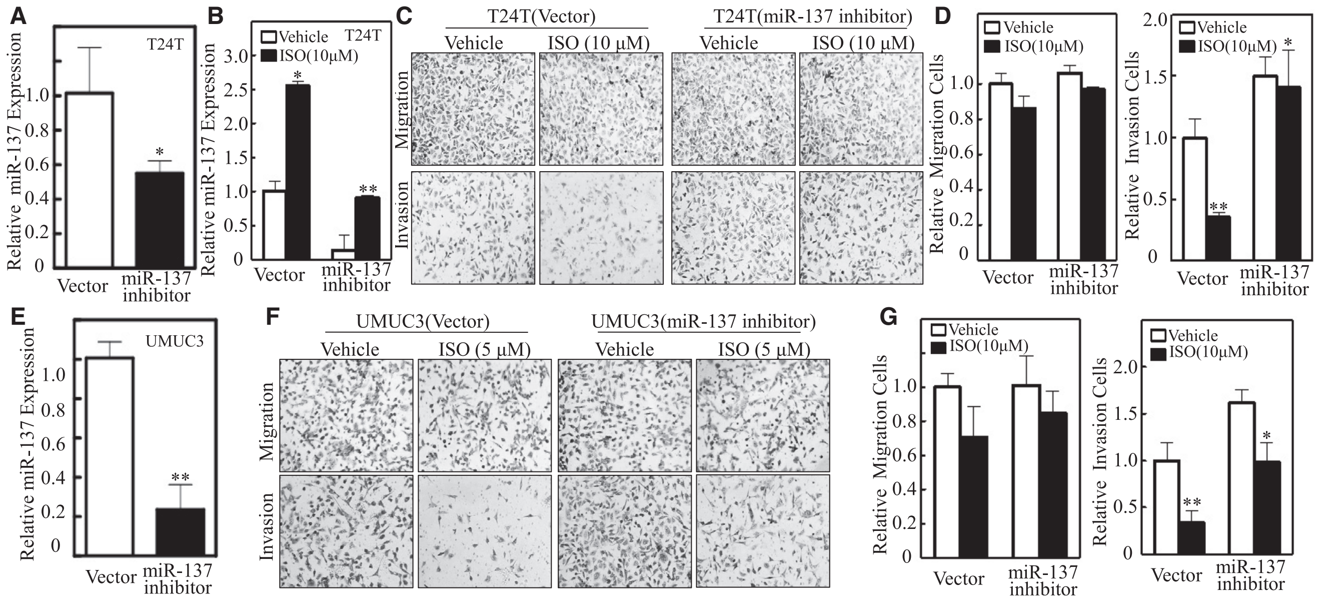

Figure 2. ISO Inhibition of BC Invasion Was Mediated by miR-137 in Both T24T and UMUC3 Cells

(A–G) Confirmation of miR-137 inhibition by qRT-PCR in T24T (A) and UMUC3 (E) cells stably transfected with has-miR-137 inhibitor (HmiR-AN0175-AM03). The miR-137 knockdown stable transfectant and its scramble control vector transfectant were subjected to cell migration and invasion assay in the presence of either vehicle or 10 μM ISO for T24T cells (C) or 5 μM ISO for UMUC3 cells (F) for 24 hr, and the relative miR-137 expression was obtain in T24T (B) with the same treatment. The migration and invasion rates in T24T (D) and UMUC3 (G) were normalized with the insert control, according to the manufacturer's instruction. The values shown are mean ± SD (n = 3). *p < 0.05; **p < 0.01.

adhesion,[23] further resulting in the suppression of cell invasion in colorectal cancer cells[24] and renal carcinoma cells,[25] whereas other studies showed that GSK3β mediates the invasion of non-invasive human lung cancer,[26] breast cancer,[27] and pancreatic cancer.[28] To explore the role of GSK3β in miR-137 inhibition of BC invasion, GSK3β expression in T24T or UMUC3 cells overexpressing miR-137 was examined (Figure 3A; Figure S1A). MiR-137 overexpression led to a remarkable inhibition of GSK3β expression in both T24T and UMUC3 cells. In contrast, after inhibiting miR-137 expression, the expression of GSK3β was upregulated in both T24T and UMUC3 cells (Figure 3B; Figure S1B). Consistently, the promotion of GSK3β in miR-137 inhibitor-overexpressed cells also increased cell-invasive abilities compared to scramble vector pGIPZ transfectant, while knockdown of GSK3β completely abolished the increased BC invasion as compared to its empty vector pSuper transfectant (Figures 3C–3E; Figure S1C). Thus, our results strongly indicate that GSK3β is an miR-137 downstream mediator responsible for BC invasion in both T24T and UMUC3 cells.

miR-137 Repressed GSK3β by Directly Interacting with Its mRNA 3′ UTR

To investigate the mechanism by which miR-137 regulated GSK3β expression, the effect of miR-137 overexpression on GSK3β mRNA expression levels in T24T and UMUC3 cells was determined. As shown in Figure 4A, there were no significant differences of GSK3β mRNA levels between T24T miR-137 and T24T vector cells. The similar expression pattern was also observed in UMUC3 miR-137 and UMUC3 vector cells (Figure 4A). To determine the relationship between the miR-137 and GSK3β expression in BC cells, three bioinformatics software, miRcode (http://www.mircode.org), miRWalk (http://mirwalk.umm.uni-heidelberg.de), and TargetscanHuman 6.2 (www.targetscan.org/vert_61) database, were utilized to screen the possible miR-137-targeted genes. The results revealed the sequences located at bases 4,268–4,274 in GSK3β 3′ UTR (NM_001146156), which were highly complementary with the seed sequence of miR-137 (Figure 4B).

Thus, we next constructed GSK3 mRNA 3′ UTR-luciferase reporter, which was co-transfected into T24T miR-137 versus T24T vector cells or T24T miR-137 inhibitor versus T24T vector cells. The results showed that miR-137 overexpression abolished GSK3β mRNA 3′ UTR activities (Figure 4C), whereas the introduction of miR-137 inhibitor remarkably increased GSK3β mRNA 3′ UTR activities (Figure 4D). Moreover, the corresponding miR-137-binding site mutation in GSK3β mRNA 3′ UTR-luciferase reporter (mut-GSK3β-4268) was also constructed, as shown in Figure 4B, and the constructs were co-transfected into T24T miR-137 versus T24T vector cells. The results indicated that, in contrast to miR-137's profound inhibition of wild-type (WT) GSK3β luciferase reporter, miR-137 did not show its suppression in mutant GSK3 reporter transfectants (Figure 4E). These results demonstrate that miR-137 represses GSK3β expression via binding to the 3′ UTR region of GSK3β mRNA.

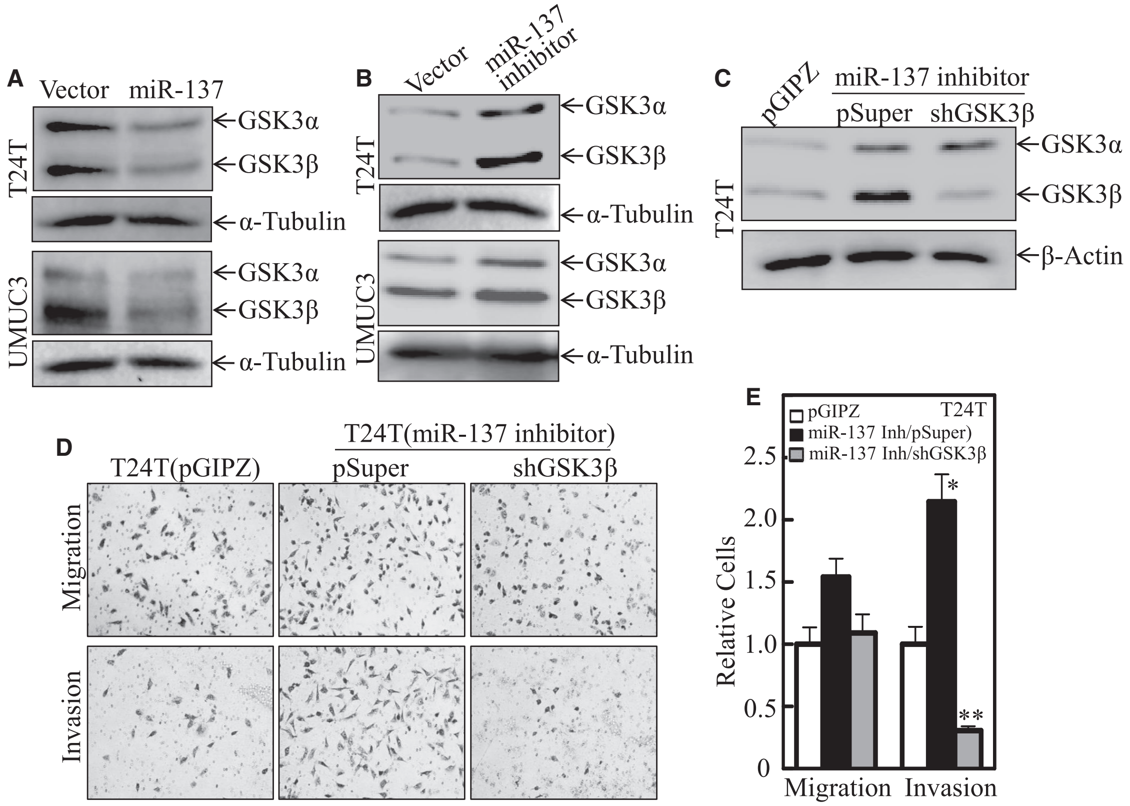

Figure 3. MiR-137 Inhibited BC Invasion by Attenuating GSK3β

(A and B) The expression of GSK3β was determined by western blotting in T24T and UMUC3 cells in the presence of miR-137 overexpression (A) or miR-137 knockdown (B). α-Tubulin was used as a loading control. (C) The cell extracts from T24T cell stable transfectants, including T24T pGIPZ, T24T miR-137-inhibitor/pSuper, and T24T miR-137-inhibitor/shGSK3β, were subjected to western blot for the determination of GSK3β protein expression, and β-actin was used as the protein loading control. (D and E) Migration and invasion abilities of T24T pGIPZ, T24T miR-137-inhibitorp/Super, and T24T miR-137-inhibitor/shGSK3β cells were determined by using the same method described above. Data are presented as mean ± SD from three independent experiments. $^{*}p < 0.05$; $^{**}p < 0.01$.

MMP-2 Was an miR-137 Downstream Effector Responsible for Its Suppression of BC Invasion

To further investigate the role of miR-137 in our experimental setting on BC cell invasion, a panel of cancer invasion-related proteins in the cells ectopically expressing miR-137 (Figure 5A) or deletion of miR-137 (Figure 5B) was analyzed by western blot. As shown in Figures 5A and 5B, WAVE3, a positive regulator of cancer cell invasion, was upregulated regardless of the increase or inhibition of miR-137 in the cells, excluding its possible involvement in miR-137 inhibition of BC invasion. Our most recent study demonstrated that RhoGDIβ plays an important role in the promotion of BC invasion.[29] The results shown in Figures 5A and 5B indicated that miR-137 overexpression promoted RhoGDIβ expression, while inhibition of miR-137 by its inhibitor abolished RhoGDIβ expression, suggesting that RhoGDIβ is not involved in miR-137 inhibition of BC invasion.

The matrix metalloproteinases (MMPs) are crucial in promoting cancer cell invasion by degrading cellular matrix components and the basement membrane,[30] and our previous work showed that MMP-2 is crucial for BC invasion.[31] Thus, we tested whether MMP-2 levels were affected by ISO treatment or miR-137 levels. The results indicated that MMP-2 protein expression was remarkably downregulated by miR-137 and upregulated by miR-137 inhibitor in T24T cells (Figures 5A and 5B; Figures S2A and S2B). Consistently, ISO treatment also attenuated MMP-2 abundance in both T24T and UMUC3 cells (Figure 5C; Figure S2C). The results from RT-PCR showed that miR-137 inhibited MMP-2 mRNA abundance (Figure 5D). These results reveal that miR-137 exhibits an inhibitory effect on MMP-2 level in human BC cells. Given that ISO treatment induces miR-137 expression and inhibits BC invasion, our results reveal that ISO suppresses BC cell invasion by elevating miR-137 expression in human BC cells.

HSP70 Was a Downstream Mediator for miR-137 Suppression of MMP-2 Expression and BC Invasion

Our above results suggest that the amount of MMP-2 mRNA was decreased in the cells overexpressing miR-137. Heat shock protein-70 (HSP70) has been reported to regulate the activity of MMPs;[32] therefore, HSP70 protein was evaluated in T24T cells ectopically expressing miR-137 or miR-137 inhibitor by immunoblotting

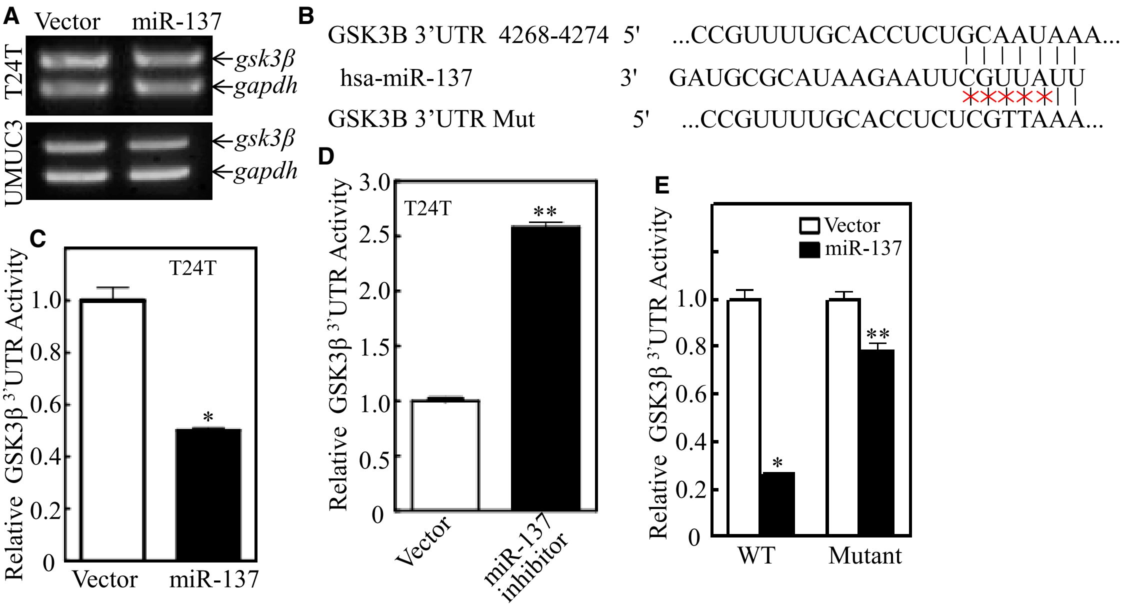

Figure 4. MiR-137 Inhibited GSK3β Protein Translation by Directly Targeting GSK3β mRNA 3′ UTR

(A) The expression levels of GSK3β mRNA were determined by RT-PCR in T24T miR-137 and UMUC3 miR-137 in comparison to their vector transfectants as indicated. (B) Schematic representation of the putative miR-137 target sites and mutant sites in 3′ UTR of GSK3β mRNA. (C and D) T24T miR-137 (C) or T24T miR-137 inhibitor (D) cells were transfected with 1 μg GSK3β mRNA 3′ UTR-luciferase reporter plasmid for each well of a 6-well plate, and the cells were then extracted for the determination of miR-137 regulation of GSK3β 3′ UTR activity. (E) T24T miR-137 and T24T vector were transfected with 1 μg of either GSK3β-3′ UTR-WT or GSK3β-3′ UTR-MUT reporter plasmid for each well of a 6-well plate, and the transfectants were subjected to luciferase activity assay. The results were presented as a relative GSP3β 3′ UTR activity with mean ± SD from three independent experiments. *p < 0.05; **p < 0.01.

analysis. The results indicated that HSP70 expression was downregulated in the cells overexpressing miR-137 in T24T cells (Figure 5E; Figure S2D), while the introduction of miR-137 inhibitor remarkably increased HSP70 abundance (Figure 5F; Figure S2E). These results consistently suggest that miR-137 does exhibit an inhibitory effect on HSP70 expression in BC cells. Moreover, knockdown of HSP70 did show a profound inhibition of MMP-2 expression and BC cell invasion, without affecting cell migration, in T24T cells (Figures 5G–5I; Figure S2F). Highly significant, ectopic expression of MMP-2 in T24T shHSP70 cells completely recused the MMP-2 expression and BC invasion (Figures 5G–5I). We next observed the effect of miR-137 inhibitor on the expression of HSP70 and MMP-2, as well as cell invasion in BC cells. As expected, knockdown of miR-137 by its inhibitor promoted the expression of HSP70 and MMP-2 in both T24T and UMUC3 cells (Figures 5J and 5K; Figures S2G and S2H). Consistent with its promotion of MMP-2 levels, the introduction of miR-137 inhibitor did specifically promote cell invasion, and such increased cell invasion by miR-137 inhibitor could be completely abolished by the knockdown of HSP70 (Figures 5L and 5M). These results strongly indicated that HSP70 is an miR-137 downstream mediator responsible for the suppression of MMP-2 expression and BC invasion.

GSK3β Was Crucial for miR-137 Promotion of HSP70 Protein Translation in T24T Cells

The above results demonstrated that miR-137 inhibition of human BC invasion is through downregulating MMP-2 in an HSP70-dependent fashion. To determine the potential influence of GSK3β on HSP70 abundance in T24T and UMUC3 cells, we examined the expression of HSP70 or MMP-2 after silencing GSK3β in the cells with or without the introduction of miR-137 inhibitor (Figures 6A and 6B; Figures S3A and S3B). As shown in Figures 6A and 6B, remarkable increased expression of HSP70 and MMP-2 was observed in either of the T24T and UMUC3 cells with suppression of miR-137 by its inhibitor. Such increased HSP70 and MMP-2 was profoundly suppressed by the knockdown of GSK3β (Figures 6A and 6B; Figures S3A and S3B).

Next, we examined the effect of GSK3β on HSP70 mRNA abundance using RT-PCR in T24T cells co-transfected with miR-137 inhibitor and small hairpin RNA (shRNA) targeting GSK3β (Figure 6C). The results showed that HSP70 mRNA level was not altered by knockdown of GSK3β, indicating that GSK3β plays no role in HSP70 gene expression at either a transcriptional or mRNA stability level (Figure 6C). The possibility of a GSK3β effect on HSP70 protein degradation was further explored. The cells expressing or not expressing shGSK3β were pre-treated with MG132 (a proteasome inhibitor) to accumulate HSP70 protein, and the cells were then subjected to HSP70 protein degradation evaluation in the presence of cycloheximide (CHX). The results indicated that HSP70 degradation rates remained similar in the cells, regardless of the presence or absence of GSK3β (Figure 6D; Figure S3C).

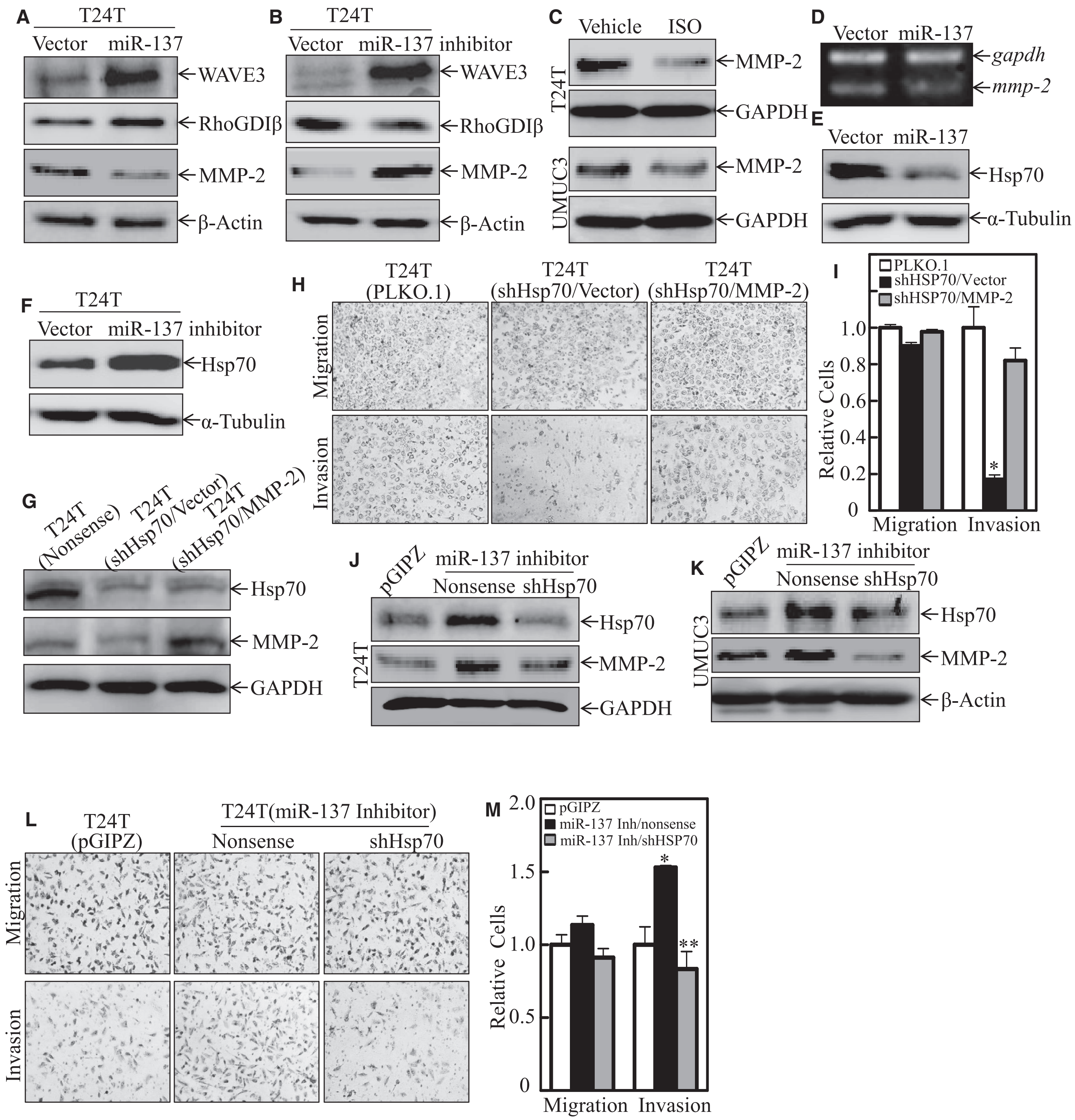

Figure 5. HSP70-MMP-2 Acted as MiR-137 Downstream Mediator and/or Effector for ISO Inhibition of BC Invasion

(A and B) The cell extracts from T24T miR-137 (A) or T24T miR-137 inhibitor (B) were subjected to western blot to determine the protein expression of WAVE3, RhoGDIβ, and MMP-2. β-Actin was used as a protein loading control. (C) T24T and UMUC3 cells were treated with either vehicle or ISO as indicated for 12 hr, and the expression of MMP-2 was determined by western blotting. GAPDH was used as a protein loading control. (D) Total RNAs were prepared from T24T vector and T24T miR-137, and then subjected to RT-PCR analyses for determining MMP-2 mRNA expression. (E and F) HSP70 protein level in T24T vector versus T24T miR-137 (E) or T24T vector versus T24T miR-137 inhibitor (F) was evaluated by western blotting, and α-tubulin was used as a loading control. (G) The cell extracts from T24T nonsense, T24T shHSP70/vector, and T24T shHSP70-MMP-2 transfectants were subjected to western blot for the determination of HSP70 and MMP-2 protein expression. GAPDH was used as a loading control. (H and I) Migration and invasion abilities of MMP-2 overexpression in T24T shHSP70 cells and the control cells were determined. Data are presented as mean ± SD from three

(legend continued on next page)

To explore the possibility that GSK3β might affect HSP70 at a translational level, the protein translation regulators, such as AKT, S6, and mTOR, and their phosphorylation statuses in T24T miR-137 inhibitor/pSuper cells were determined in comparison to those in T24T miR-137 inhibitor/shGSK3β cells (Figure 6E; Figure S3D). The total protein levels of AKT, S6, and mTOR were comparable, whereas the phosphorylation levels of these protein were dramatically decreased in T24T miR-137 inhibitor/shGSK3β cells. To provide direct evidence showing GSKβ regulation of HSP70 protein translation, the new HSP70 protein-synthesizing rates were evaluated by using ^{35}S-methionine/cysteine pulse-labeling assay in T24T miR-137 inhibitor/pSuper versus T24T miR-137 inhibitor/shGSK3β cells (Figure 6F). The incorporation of ^{35}S-labeled HSP70 protein was gradually elevated along with the incubation time periods in the scramble control T24T miR-137 inhibitor/pSuper cells, whereas such newly synthesized HSP70 was attenuated in T24T miR-137 inhibitor/shGSK3β cells. Collectively, these data clearly reveal that GSK3β is crucial for miR-137 promotion of HSP70 protein translation in T24T cells.

ISO Treatment Upregulated miR-137 through Promoting c-Jun Phosphorylation

To elucidate the mechanisms leading to the elevation of miR-137 abundance due to ISO treatment, miR-137 expression and miR-137 promoter activity in ISO-treated T24T cells were determined. ISO treatment increased both miR-137 expression and its promoter activity (Figures 7A and 7B), suggesting that ISO promotes miR-137 abundance at a transcriptional level. To identify the transcription factor(s) that is/are responsible for ISO-induced miR-137 upregulation, TFANSFAC Transcription Factor Binding Sites Software (Biological Database, Wolfenbutel, Germany) was used for bioinformatics analysis of the miR-137 promoter region. The results revealed that the putative DNA-binding sites of various transcription factors, including Jun D, HSF1, c-Jun, c-Fos, and E2F1, are located at an approximate 2,000-bp promoter region of human miR-137 (Figure 7C). The expressions of these transcription factors in T24T and UMUC3 cells were further examined after ISO treatment.

As shown in Figures 7D and 7E and Figures S4A and S4B, the levels of c-Jun phosphorylated at Ser73, Jun D, HSF1, c-Fos, and E2F1 were markedly increased in ISO-treated T24T and UMUC3 cells. Since Jun D has been reported to always inhibit its targeted gene transcription, we anticipated Jun D might not participate in ISO induction of miR-137. Thus, the constructs expressing E2F1, HSF1, or a dominant-negative mutant form of c-Jun (TAM67) were stably transfected into T24T cells, and the effects of them on the expression of MMP-2 and miR-137 were further assessed. The results indicated that ectopic expression of either HSF1 or E2F1 did not show any observable inhibitory effect on MMP-2 expression and had no effect on miR-137 expression in T24T cells, excluding their roles in ISO induction of miR-137 transcription (Figures 7F–7I; Figures S4C and S4D). Interestingly, the inhibition of c-Jun activation by ectopic expression of TAM67 in T24T cells slightly reduced miR-137 level in untreated cells (Figures 7J and 7K; Figure S4E) and significantly decreased ISO-induced miR-137 abundance (Figure 7L). Consistently, ISO-induced increases in c-Jun phosphorylation at Ser73 and MMP-2 and decreases in GSK3β and GSK3β were completely blocked in T24T TAM67 cells (Figure 7M; Figure S4F), indicating that suppression of c-Jun activation by TAM67 could mimic ISO treatment. Thus, it appears that active c-Jun acts as a transcription factor binding to the miR-137 promoter and initiates miR-137 transcription, as illustrated in Figure 7N.

DISCUSSION

Muscular invasion BC causes almost 100% death, which means a serious therapeutic challenge of this disease. Thus, to discover new anti-cancer compounds with efficient inhibitory effect on BC invasion is extremely important and urgent. Our current finding of miR-137 suppression of MMP-2 expression, in conjunction with our previous discovery that ISO, via the induction of miR-137, inhibited BC invasion and BC growth *in vitro* and *in vivo*,[7,12] releases the nature of ISO as a potent BC therapeutic agent.

As a new derivative of stilbene compound discovered to affect the competence of cancers, ISO at relevant applicable concentrations also effectively abolishes transcription factor specific protein 1 (Sp1) expression and transactivation, leading to the downregulation of bindings of Sp1 to the promoter region of its regulated genes, cyclin D1 and XIAP, and further suppression of cancer cell anchorage-independent growth and induction of apoptosis in BC cells.[10,12] In our published study, ISO was also reported to specifically suppress BC invasion through targeting the STAT1-FOXO1-MMP-2 axis.[7] *In vitro*, ISO suppresses human BC cell invasion, accompanied by upregulation of the forkhead box class O 1 (FOXO1) mRNA transcription.[7] The transcription level of MMP-2 is also inhibited by FOXO1 following ISO treatment *in vitro*,[7] and this finding *in vitro* is consistently supported by data obtained from an *in vivo* mouse model of ISO inhibition, BBN-induced mouse-invasive BC formation.[7] In our current study, the invasion ability of both T24T and UMUC3 cells, treated with relevant applicable concentrations of ISO, is significantly and specifically attenuated, while there is no significant difference between ISO treatment and the vehicle control in migration ability (data that are consistent with the results we reported previously[7]), reflecting that, both *in vivo* and *in vitro*, ISO is an efficient anti-cancer agent for the specific inhibition of BC invasion.

MiR-137 is located on human chromosome 1p22, and it was found to mediate in ISO the induction of G0-G1 growth arrest in BC cells.[12]

independent experiments. (J and K) The cell extracts from T24T miR-137-inhibitor/vector and its control vector transfectant T24T pGIPZ, as well as T24T miR-137-inhibitor/shHSP70 (J), UMUC3 pGIPZ, UMUC3 miR-137-inhibitorion/vector, and UMUC3 miR-137-inhibitor/shHSP70 cells (K), were subjected to western blot for the determination of HSP70 and MMP-2 protein expression. (L and M) Migration and invasion abilities of T24T pGIPZ, T24T miR-137-inhibitor/vector, and T24T miR-137-inhibitor/shHSP70 cells were determined. Data are presented as mean ± SD from three independent experiments. *$p < 0.05$; **$p < 0.01$.

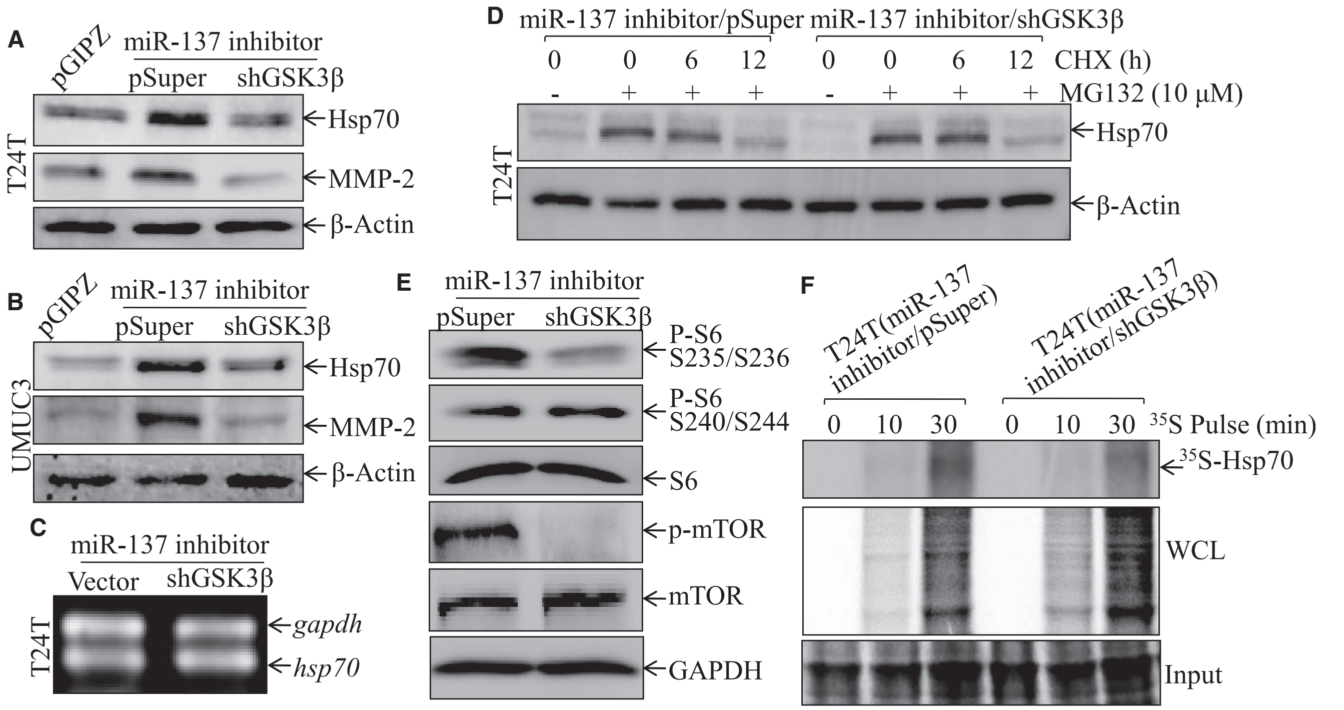

Figure 6. GSK3β Played a Key Role in HSP70 Translation and MMP-2 Protein Expression

(A and B) The cell extracts from T24T pGIPZ, T24T miR-137-inhibitor/vector, T24T miR-137-inhibitor/shGSK3β, UMUC3 pGIPZ, UMUC3 miR-137-inhibitor/vector, and UMUC3 miR-137-inhibitor/shHSP70 cells were subjected to western blot for the determination of HSP70 and MMP-2 protein expression. (C) The mRNA expression levels of HSP70 were determined by RT-PCR in T24T miR-137-inhibitor/shHSP70 cells in comparison to T24T miR-137-inhibitor/vector cells. (D) T24T miR-137-inhibitor/vector and T24T miR-137-inhibitor/shGSK3β cells were treated with MG132 for 4 hr, followed by cycloheximide (CHX) for the indicated time points. The cell extracts were subjected to western blotting, and β-actin protein expression was used as a protein loading control. The result was a representative one from three independent experiments. (E) The cell extracts from T24T miR-137-inhibitor/vector and T24T miR-137-inhibitor/shGSK3β transfectants were subjected to western blot for the determination of protein expression as indicated. (F) Newly synthesized HSP70 protein was monitored by pulse assay using ^{35}S-labeled methionine/cysteine in the indicated transfectants (WCL, whole-cell lysate). Coomassie blue staining was used for a protein loading control, as described in the Materials and Methods.

Similar to ISO treatment, ectopic expression of miR-137 alone leads to G0-G1 cell growth arrest and inhibition of anchorage-independent growth in human BC cells, which in turn suppresses Sp1 protein translation by directly targeting Sp1 mRNA 3′ UTR.[12] Moreover, the G0-G1 cell growth arrest and inhibition of anchorage-independent growth by ectopic expression of miR-137 could be completely reversed by overexpression of GFP-Sp1.[12] Our published studies also demonstrate that miR-137 is profoundly downregulated in human BC patients and mouse highly invasive BCs, while ISO treatment remarkably elevates miR-137 in ISO-treated BC cells.[12] However, the mechanisms leading to miR-137 induction and function in the ISO inhibition of BC invasion were never explored. In this study, we demonstrate that ISO at relevant concentrations of 5–10 μM represses BC invasion through promoting c-Jun activation and further upregulating miR-137 transcription. We also show that ectopic expression of miR-137 exhibits a great inhibitory effect on BC-invasive ability, suggesting potential application of miR-137 as a therapeutic value in new drug screening and invasive BC therapy as well.

C-Jun is a transcription factor that is activated via phosphorylation at Ser63 and Ser73.[33,34] We have demonstrated that ISO treatment induces c-Jun phosphorylation and further induces SESN2 transcription and cell autophagy in BC cells.[11] Consistently, we show here that ISO treatment upregulated miR-137 through promoting the phosphorylation of c-Jun at Ser73, consequently resulting in the inhibition of GSK3β-HSP70 protein translation, MMP-2 protein elevation, and BC cell invasion. Our results also reveal that ISO treatment significantly increased c-Jun phosphorylation without affecting its total protein level, indicating that ISO might activate the c-Jun N-terminal kinases. Nevertheless, the underlying mechanism of ISO action on c-Jun phosphorylation is currently under investigation.

Extensive studies have identified MMPs as the key players in cancer cell invasion by degrading cellular matrix components and the basement membrane.[30] Elevated expression levels of MMP-2 (collagenase type IV, one of the MMP family members) have been reported to be of independent prognostic value in patients with BCs,[35] correlate with the risk of recurrence,[36,37] and promote cancer progression by allowing cancer cells to migrate from tumor *in situ* and metastasize to other organs.[38] MMP-2 transcriptional regulation is part of a delicate balance between the expression of various extracellular matrix (ECM) constituents and ECM-degrading enzymes, and the transcription

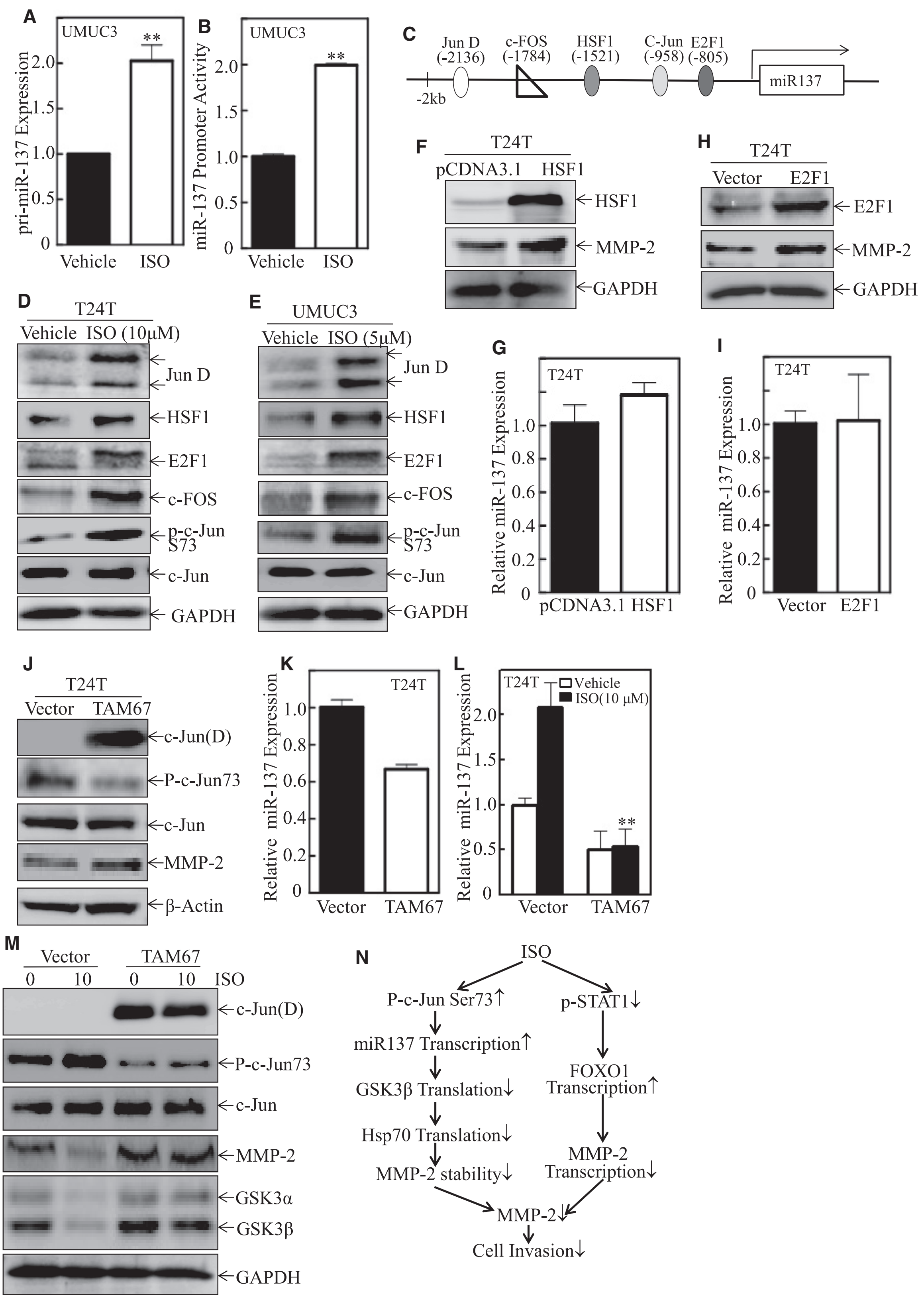

(legend on next page)

rate of MMP-2 in BC has been shown to be regulated by Ets1,[39] Sp1,[40] p-Erk,[41] and FOXO1[7] in several previous publications. p38 MAPK and its downstream effector, MAPKAPK2, regulate MMP-2 by stabilizing their mRNA transcripts in BC cells.[42] Although MMP-2 post-transcriptional level has been reported to be induced by transforming growth factor β (TGF-β)2 through independent of phosphatidylinositol 3-kinase-signaling pathway,[43] few studies focus on the regulation of MMP-2 mRNA stability. In this study, we find that miR-137 inhibits BC cell invasion by downregulating MMP-2 in both T24T and UMUC3 cells through the inhibition of HSP70 protein translation. Several lines of evidence suggest that heat shock proteins are associated with the regulation of RNA metabolism. The binding of HSP70 to its own mRNA causes the rapid alterations in mRNA stability.[44] Thus, it is conceivable that HSP70 upregulates MMP-2 protein by promoting its mRNA stabilization directly, and, therefore, further study of this notion is currently underway in our laboratory.

With regard to the mechanism by which miR-137 regulates the MMP-2 and, subsequently, causes the suppression of cancer invasion by ISO treatment, we discover that GSK3β plays an essential role in mediating these observed effects. GSK3β is a ubiquitously expressed serine/threonine protein kinase that is important for establishing chemo- or radio-resistance in cancers.[45] In this process, GSK3β directly phosphorylated β-catenin, cyclin D1, and cyclin D2 for them to be ubiquitilated and subsequently degraded.[46] GSK3β is reported to be implicated in promoting cancer invasion.[47] We demonstrate here that miR-137 is able to bind to the 3′ UTR of GSK3β mRNA, suppresses GSK3β protein translation, and, in turn, influences the expression of HSP70 and MMP-2 (Figure 7N). It is noted that GSK3β exerts the positive regulatory effect on the HSP70 protein expression in BC cells. However, the levels of HSP70 in T24T cells with or without GSK3β are comparable after being pre-treated with proteasome inhibitor, suggesting that GSK3β-promoted HSP70 expression is not regulated by the proteasomal degradation system. We further demonstrate that GSK3β activates the phosphorylation of protein translation-related machinery, such as mTOR and S6, and upregulates HSP70 protein translation, thereby increasing HSP70-MMP-2 protein expression and BC invasion.

In summary, our results reveal that ISO treatment results in c-Jun phosphorylation/activation, and the activated c-Jun binds to the miR-137 promoter region, resulting in the promotion of miR-137 transcription, which consequently inhibits GSK3β and HSP70 protein translation, MMP-2 mRNA stability, and BC invasion. Considering that miR-137 is downregulated in many cancer tissues, including BCs, this novel p-c-Jun/miR-137-GSK3β-HSP70-MMP-2 axis would provide significant information to explore a potential therapeutic strategy for patients with invasive BC.

MATERIALS AND METHODS

Cell Culture and Transfections

UMUC3 and T24T cells were human high-grade invasive BC cells and used in our previous studies.[11,48] These cells and their stable transfectants were maintained at 37°C in a 5% CO_2 incubator with DMEM supplemented with 10% fetal bovine serum (FBS), 2 μM L-glutamine, and 25 μg/mL gentamycin. The monolayer growth of human BC T24T cells was maintained in DMEM-F12 (1:1) (Invitrogen), supplemented with 5% heat-inactivated FBS, 2 μM L-glutamine, and 25 μg/mL gentamycin, as described in previous studies.[7,31] All cell lines were subjected to DNA tests and authenticated before and after utilization for research by Genetica DNA Laboratories (Burlington, NC, USA) using a PowerPlex 16 HS System. Cell transfections were performed by using PolyJet DNA *In Vitro* Transfection Reagent (SignaGen Laboratories, Rockville, MD, USA) together with 1 μg of each plasmid for each well of a 6-well plate, according to the manufacturer's instructions. Surviving cells from the antibiotics selection were pooled as stable mass transfectants, as described in our previous studies.[10,31]

Reagents, Plasmids, and Antibodies

The dual luciferase assay kit, TRIzol reagent and SuperScript First-Strand Synthesis system were purchased from Promega (Madison, WI, USA) and Invitrogen (Grand Island, NY, USA), respectively. PolyJet DNA *In Vitro* Transfection Reagent was bought from SignaGen Laboratories (Rockville, MD, USA). The luciferase assay substrate was from Promega (Madison, WI, USA). MG132 and CHX were purchased from Calbiochem (San Diego, CA, USA). ISO with purity more than 99% was purchased from Rochen Pharma

Figure 7. ISO Induced MiR-137 Transcription by Promoting c-Jun Phosphorylation at Ser73

(A) The expression levels of pri-miR-137 were determined by real-time PCR in UMUC3 with 5 μM ISO treatment for 12 hr. (B) UMUC3 cells were transfected with miR-137 promoter-driven luciferase reporter together with pRL-TK, and then they were treated with ISO or vehicle control for the determination of miR-137 promoter transactivation. The luciferase activity was assessed, and pRL-TK was used as an internal control to normalize the transfection efficiency. The two asterisks indicate a significant increase as compared with vehicle-treated UMUC3 cells ($p < 0.01$). (C) Potential transcriptional factor-binding sites in miR-137 promoter region (–2,000–+1) analyzed by using the ALGGEN engine online. (D and E) T24T (D) and UMUC3 (E) cells were treated with either vehicle or ISO for 12 hr. Expression of the related transcription factors in the whole-cell lysates was determined by western blotting, and GAPDH was used as a protein loading control. (F and H) T24T cells were stably transfected with HSF1-, E2F1-, and TAM67-expressing plasmids, respectively. HSF1 (F) and E2F1 (H) overexpression in T24T was determined by western blot. GAPDH was used as a loading control. (G and I) The total miRNA was extracted from each cell line and miR-137 was evaluated by real-time PCR. (J) TAM67 overexpression in T24T was assessed by western blot. Expression of c-Jun (D), c-Jun, p-c-Jun73, and MMP-2 in the whole-cell lysates was determined by western blot, and β-actin was used as a loading control. (K) The miRNA was extracted from T24T cells stably transfected with TAM67 and its scramble vector, and miR-137 expression was examined. (L and M) Treated with medium containing either vehicle or 10 μM ISO for 12 hr, T24T TAM67, the stable T24T vector, and cells were subjected to miR-137 expression (L), and the relative protein expression (M) was identified by western blot. GAPDH was used as a loading control. (N) The proposed schematic for the cascade underlying ISO inhibition of human BC cell invasion through the downregulation of MMP-2 in a c-Jun/miR-137/GSK3β/ HSP70-dependent axis.

(Shanghai, China) and was dissolved in DMSO to make a stock concentration at 20 mM.

The 3′ UTR of GSK3β mRNA was cloned into the p-MIR luciferase reporter vector. In brief, GSK3β 3′ UTR-1 (1,446–1,452) was amplified by PCR using the following primer set: 5′-CCG CTC GAG TGA AAA TTG AGC TTG CAG AA-3′(forward) and 5′-CCC AAG CTT CAC AGT TAA GGA GCA GGA CA-3′(reverse). GSK3β 3′ UTR-2 (4,268–4,274) was amplified by PCR using the following primer set: 5′-CCG CTC GAG CAC TGG CAT TTC ATC TAT TT-3′ (forward) and 5′-CTA GAC TAG T AA GTG GTC ACG CTA ATT GGT ATG-3′ (reverse). The amplified fragment was subcloned into p-MIR luciferase reporter vector. Each mutation's plasmid, constructed in the miR-137-binding site in p-MIR-GSK3β, was introduced by using fusion PCR and the following primers: GSK3β 3′ UTR-1-mut, 5′-TCT CTC TTT TTG AAG AAA ATC GTA TAT TCC TTG GAA AGC AAG-3′ and 5′-CTT GCT TTC CAA GGA ATA TAC GAT TTT CTT CAA AAA GAG AGA-3′; GSK3β 3′ UTR-2-mut, 5′-CTC CGT TTT GCA CCT CTC GTT TAA AAG CAA AAT GAC AA-3′ and 5′-TTG TCA TTT TGC TTT TAA ACG AGA GGT GCA AAA CGG AG-3′. The miR-137 expression construct, pcDNA3.2/V5-mmu-miR-137, was obtained from Addgene (Cambridge, MA, USA). The miR-137 inhibitor expression plasmid (HmiR-AN0175-AM03) was purchased from Gencopoeia (Rockville, MD, USA). The GSK3β shRNA construct (psuper-neo-GFP) was used in our previous study,[49] and the plasmid of shRNA specifically targeting HSP70 was purchased from Open Biosystems (Huntsville, AL, USA) with the hairpin sequence: (1) 5′-CCG GGC TGA CGA AGA TGA AGG AGA TCT CGA GAT CTC CTT CAT CTT CGT CAG CTT TTT-3′, and (2) 5′-GGG AAC CCG CAG AAC ACC GTG TTC TCG AGA ACA CGG TGT TCT GCG GGT TCT TTT T-3′. The plasmid TAM67, a dominant-negative mutant of c-Jun, was described previously.[50] Antibodies against c-Jun(D), HSP70, GSK3β, p-c-Jun S73, S6, p-S6 S235/236, p-S6 S240/244, p-Akt S473, p-Akt T308, and Akt were bought from Cell Signaling Technology (Beverly, MA, USA); anti-β-actin was purchased from Sigma (St. Louis, MO, USA); and antibodies against MMP-2, E2F1, HSF1, and GAPDH were purchased from Santa Cruz Biotechnology (Santa Cruz, CA, USA).

RT–PCR and Real-Time qPCR

Total RNA was extracted with TRIzol reagent, followed by RNA precipitation with isopropyl alcohol and purification with 75% ethanol, according to the manufacturer's instructions. Total RNA (5 μg) was used for first-strand cDNA synthesis with oligdT primer by SuperScript First-Strand Synthesis system (Invitrogen). Specific primer pairs were designed for amplifying the following: human GSK3β (forward: 5′-AGC TCC AGA TCA TGA GAA AG-3′, reverse: 5′-GAC CAG CTG CTT TGC ACT TC-3′), human HSP70 (forward: 5′-CAA CAC GGC AAG GTG GAG ATC A-3′, reverse: 5′-TCA GCC GCT TCG CGT CAA ACA-3′), human MMP-2 (forward: 5′-CAA GTG GGA CAA GAA CCA GA-3′, reverse: 5′-CCA AAG TTG ATC ATG ATG T-3′), human GAPDH (forward: 5′-GAT GAT CTT GAG GCT GTT GTC-3′, reverse: 5′-CAG GGC TGC TTT TAA CTC TG-3′), human pri-miR-137 (forward: 5′-CTC TTC GGT GAC GGG TAT-3′, reverse: 5′-CAA TAA CAA CGT AAT CCG-3′), and miR-137 (5′-TAT TGC TTG AGA ATA CAC GTA G-3′).

Western Blot Analyses

UMUC3 and T24T cells as well as their transfectants were seeded in six-well plates and cultured in normal culture medium until 70%–80% confluence. Whole-cell extracts were then prepared with the cell lysis buffer (10 mM Tris-HCl [pH 7.4], 1% SDS, and 1 mM Na_3VO_4), as described in our previous study.[50] Cell extracts were subjected to western blot analysis, and the protein bands specifically bound to the primary antibodies were detected using an alkaline phosphatase-linked secondary antibody and an ECF Western Blot system (Amersham, Piscataway, NJ, USA), as described previously.[50,51] The images were acquired by scanning with the phosphor imager (Typhoon FLA 7000, GE, Pittsburgh, PA, USA). Western blotting experiments were repeated at least three times and the representative blots are shown in the figures. The densitometry analyses of the specific protein band relative to loading control protein were performed using ImageJ (NIH, Bethesda, MD, USA). Results were presented as the means ± SD of triplicates.

Cell Migration and Invasion Assay

Control inserts without Matrigel and the invasion kit were purchased from BD Biosciences (Bedford, MA, USA). The cells (3×10^4) were seeded onto inserts in triplicate, in 400 μL serum-free DMEM or F12-DMEM. Inserts were placed into wells containing 1 mL medium supplemented with 10% FBS. The cells were incubated for 24 hr. Cells on the upper surface of the filters were completely removed by wiping with a cotton swab. The inserts were then fixed in methanol and stained with Giemsa. The numbers of migrated cells attached to the other side of the insert were counted under a light Olympus DP71 microscope in eight random fields at a magnification of ×200. The number of migrated and invasive cells per image was determined using ImageJ software. The data shown are representative of three independent experiments.

Luciferase Assay

The miR-137 overexpression or inhibitor constructs were transfected into T24T cells together with the renilla luciferase vector pRL-TK and GSK3β 3′ UTR-WT luciferase reporter or its mutant luciferase reporter. At 48 hr after transfection, the cells were extracted by using lysis buffer according to the dual-luciferase assay manual (Promega), and then they were subjected to luciferase assay using a luminometer (Lumat LB9507, Berthold Technologies, Bad Wildbad, Germany) together with the luciferase Assay System kit (Promega, Madison, WI, USA). The results were normalized by internal TK signal and presented as relative GSK3β 3′ UTR activity with mean ± SE from triplicate assays.

[^{35}S] Methionine Pulse Assays

Cells of T24T miR-137 inhibitor/pSuper and T24T miR-137 inhibitor/shGSK3β were incubated with methionine-cysteine-free DMEM

(Gibco-BRL, Grand Island, NY, USA) containing 2% dialyzed fetal calf serum (Gibco-BRL, Grand Island, NY, USA) and 50 μM MG132 for 1 hr. The cells were then incubated with 2% FBS methionine-cysteine-free DMEM containing ^{35}S-labeled methionine/cysteine (250 μCi per dish, Trans ^{35}S-label; ICN) for the indicated time periods. The cells were extracted with lysis buffer (Cell Signaling Technology, Beverly, MA, USA) containing complete protein inhibitor mixture (Roche, Branchburg, NJ, USA) on ice for 10 min. Total lysate of 500 mg was incubated with anti-HSP70 antibody-conjugated agarose beads (R&D Systems, Minneapolis, MN, USA) overnight at 4°C. The immunoprecipitated samples were washed with the cell lysis buffer five times, heated at 100°C for 5 min, and then subjected to SDS-PAGE analysis. The images of ^{35}S-labeled HSP70 protein band were acquired by scanning with the phosphor imager (Typhoon FLA 7000, GE, Pittsburgh, PA, USA).

Statistical Analysis

The Student's t test was used to determine the significant difference between treated and untreated groups. The results are expressed as mean ± SD from at least three independent experiments. $p < 0.05$ was considered as a significant difference between the compared groups.

SUPPLEMENTAL INFORMATION

Supplemental Information includes four figures and can be found with this article online at https://doi.org/10.1016/j.omtn.2018.05.017.

AUTHOR CONTRIBUTIONS

C.H. and H.H. were involved in design of the study. X.G., H.J., H.H., J.X., J.L., H.Y., X.L., X.Z., and L.X. carried out the experiments and acquired data (cell culturing, gene expression analysis, etc.). C.H., X.G., H.H., S.R., C.C., and H.J. analyzed the data and drafted the manuscript. J.L. provided administrative, technical, and material support. C.H. supervised the study and all authors reviewed the manuscript.

CONFLICTS OF INTEREST

The authors declare that they have no actual or potential competing financial interests.

ACKNOWLEDGMENTS

We thank Ms. Nedda Tichi for her critical reading of the manuscript. This work was partially supported by grants from the NIH/NCI (CA165980, CA177665, and CA217923), NIH/NIEHS (ES000260), and the Natural Science Foundation of China (NSFC81773391 and NSFC81702530).

REFERENCES

1. Jemal, A., Siegel, R., Xu, J., and Ward, E. (2010). Cancer statistics, 2010. CA Cancer J. Clin. *60*, 277–300.
2. Wu, X.R. (2005). Urothelial tumorigenesis: a tale of divergent pathways. Nat. Rev. Cancer *5*, 713–725.
3. Divrik, R.T., Sahin, A.F., Yildirim, U., Altok, M., and Zorlu, F. (2010). Impact of routine second transurethral resection on the long-term outcome of patients with newly diagnosed pT1 urothelial carcinoma with respect to recurrence, progression rate, and disease-specific survival: a prospective randomised clinical trial. Eur. Urol. *58*, 185–190.
4. Herr, H., Konety, B., Stein, J., Sternberg, C.N., and Wood, D.P., Jr. (2009). Optimizing outcomes at every stage of bladder cancer: do we practice it? Urol. Oncol. *27*, 72–74.
5. Siegel, R.L., Miller, K.D., and Jemal, A. (2016). Cancer statistics, 2016. CA Cancer J. Clin. *66*, 7–30.
6. Lotan, Y., Kamat, A.M., Porter, M.P., Robinson, V.L., Shore, N., Jewett, M., Schelhammer, P.F., deVere White, R., Quale, D., and Lee, C.T.; Bladder Cancer Think Tank; Bladder Cancer Advocacy Network; Society of Urologic Oncology (2009). Key concerns about the current state of bladder cancer: a position paper from the Bladder Cancer Think Tank, the Bladder Cancer Advocacy Network, and the Society of Urologic Oncology. Cancer *115*, 4096–4103.
7. Jiang, G., Wu, A.D., Huang, C., Gu, J., Zhang, L., Huang, H., Liao, X., Li, J., Zhang, D., Zeng, X., et al. (2016). Isorhapontigenin (ISO) Inhibits Invasive Bladder Cancer Formation In Vivo and Human Bladder Cancer Invasion In Vitro by Targeting STAT1/FOXO1 Axis. Cancer Prev. Res. (Phila.) *9*, 567–580.
8. Xu, Z., Zeng, X., Xu, J., Xu, D., Li, J., Jin, H., Jiang, G., Han, X., and Huang, C. (2016). Isorhapontigenin suppresses growth of patient-derived glioblastoma spheres through regulating miR-145/SOX2/cyclin D1 axis. Neuro-oncol. *18*, 830–839.
9. Gao, G., Chen, L., Li, J., Zhang, D., Fang, Y., Huang, H., Chen, X., and Huang, C. (2014). Isorhapontigenin (ISO) inhibited cell transformation by inducing G0/G1 phase arrest via increasing MKP-1 mRNA Stability. Oncotarget *5*, 2664–2677.
10. Fang, Y., Yu, Y., Hou, Q., Zheng, X., Zhang, M., Zhang, D., Li, J., Wu, X.R., and Huang, C. (2012). The Chinese herb isolate isorhapontigenin induces apoptosis in human cancer cells by down-regulating overexpression of antiapoptotic protein XIAP. J. Biol. Chem. *287*, 35234–35243.
11. Liang, Y., Zhu, J., Huang, H., Xiang, D., Li, Y., Zhang, D., Li, J., Wang, Y., Jin, H., Jiang, G., et al. (2016). SESN2/sestrin 2 induction-mediated autophagy and inhibitory effect of isorhapontigenin (ISO) on human bladder cancers. Autophagy *12*, 1229–1239.
12. Zeng, X., Xu, Z., Gu, J., Huang, H., Gao, G., Zhang, X., Li, J., Jin, H., Jiang, G., Sun, H., and Huang, C. (2016). Induction of miR-137 by Isorhapontigenin (ISO) Directly Targets Sp1 Protein Translation and Mediates Its Anticancer Activity Both In Vitro and In Vivo. Mol. Cancer Ther. *15*, 512–522.
13. Dalmay, T. (2013). Mechanism of miRNA-mediated repression of mRNA translation. Essays Biochem. *54*, 29–38.
14. Zhao, Y., Li, Y., Lou, G., Zhao, L., Xu, Z., Zhang, Y., and He, F. (2012). MiR-137 targets estrogen-related receptor alpha and impairs the proliferative and migratory capacity of breast cancer cells. PLoS ONE *7*, e39102.
15. Guo, J., Xia, B., Meng, F., and Lou, G. (2013). miR-137 suppresses cell growth in ovarian cancer by targeting AEG-1. Biochem. Biophys. Res. Commun. *441*, 357–363.
16. Chen, Q., Chen, X., Zhang, M., Fan, Q., Luo, S., and Cao, X. (2011). miR-137 is frequently down-regulated in gastric cancer and is a negative regulator of Cdc42. Dig. Dis. Sci. *56*, 2009–2016.
17. Chen, D.L., Wang, D.S., Wu, W.J., Zeng, Z.L., Luo, H.Y., Qiu, M.Z., Ren, C., Zhang, D.S., Wang, Z.Q., Wang, F.H., et al. (2013). Overexpression of paxillin induced by miR-137 suppression promotes tumor progression and metastasis in colorectal cancer. Carcinogenesis *34*, 803–811.
18. Yang, Y.R., Li, Y.X., Gao, X.Y., Zhao, S.S., Zang, S.Z., and Zhang, Z.Q. (2015). MicroRNA-137 inhibits cell migration and invasion by targeting bone morphogenetic protein-7 (BMP7) in non-small cell lung cancer cells. Int. J. Clin. Exp. Pathol. *8*, 10847–10853.
19. Althoff, K., Beckers, A., Odersky, A., Mestdagh, P., Köster, J., Bray, I.M., Bryan, K., Vandesompele, J., Speleman, F., Stallings, R.L., et al. (2013). MiR-137 functions as a tumor suppressor in neuroblastoma by downregulating KDM1A. Int. J. Cancer *133*, 1064–1073.
20. Luo, Y., Li, X., Dong, J., and Sun, W. (2016). microRNA-137 is downregulated in thyroid cancer and inhibits proliferation and invasion by targeting EGFR. Tumour Biol. *37*, 7749–7755.
21. Han, Y., Bi, Y., Bi, H., Diao, C., Zhang, G., Cheng, K., and Yang, Z. (2016). miR-137 suppresses the invasion and procedure of EMT of human breast cancer cell line MCF-7 through targeting CtBP1. Hum. Cell *29*, 30–36.

22. Jiang, G., Huang, C., Li, J., Huang, H., Wang, J., Li, Y., Xie, F., Jin, H., Zhu, J., and Huang, C. (2018). Transcriptional and post-transcriptional upregulation of p27 mediates growth inhibition of isorhapontigenin (ISO) on human bladder cancer cells. Carcinogenesis *39*, 482–492.

23. Yoshino, Y., Suzuki, M., Takahashi, H., and Ishioka, C. (2015). Inhibition of invasion by glycogen synthase kinase-3 beta inhibitors through dysregulation of actin re-organisation via down-regulation of WAVE2. Biochem. Biophys. Res. Commun. *464*, 275–280.

24. Zhang, H., Hou, W., Wang, H.L., Liu, H.J., Jia, X.Y., Zheng, X.Z., Zou, Y.X., Li, X., Hou, L., McNutt, M.A., and Zhang, B. (2014). GSK-3β-regulated N-acetyltransferase 10 is involved in colorectal cancer invasion. Clin. Cancer Res. *20*, 4717–4729.

25. Wei, R.J., Zhang, C.H., and Yang, W.Z. (2017). MiR-155 affects renal carcinoma cell proliferation, invasion and apoptosis through regulating GSK-3β/β-catenin signaling pathway. Eur. Rev. Med. Pharmacol. Sci. *21*, 5034–5041.

26. Wang, W.L., Hong, T.M., Chang, Y.L., Wu, C.T., Pan, S.H., and Yang, P.C. (2012). Phosphorylation of LCRMP-1 by GSK3β promotes filopoda formation, migration and invasion abilities in lung cancer cells. PLoS ONE *7*, e31689.

27. Yan, D., Avtanski, D., Saxena, N.K., and Sharma, D. (2012). Leptin-induced epithelial-mesenchymal transition in breast cancer cells requires β-catenin activation via Akt/GSK3- and MTA1/Wnt1 protein-dependent pathways. J. Biol. Chem. *287*, 8598–8612.

28. Kitano, A., Shimasaki, T., Chikano, Y., Nakada, M., Hirose, M., Higashi, T., Ishigaki, Y., Endo, Y., Takino, T., Sato, H., et al. (2013). Aberrant glycogen synthase kinase 3β is involved in pancreatic cancer cell invasion and resistance to therapy. PLoS ONE *8*, e55289.

29. Huang, H., Jin, H., Zhao, H., Wang, J., Li, X., Yan, H., Wang, S., Guo, X., Xue, L., Li, J., et al. (2017). RhoGDIβ promotes Sp1/MMP-2 expression and bladder cancer invasion through perturbing miR-200c-targeted JNK2 protein translation. Mol. Oncol. *11*, 1579–1594.

30. Gialeli, C., Theocharis, A.D., and Karamanos, N.K. (2011). Roles of matrix metalloproteinases in cancer progression and their pharmacological targeting. FEBS J. *278*, 16–27.

31. Jin, H., Yu, Y., Hu, Y., Lu, C., Li, J., Gu, J., Zhang, L., Huang, H., Zhang, D., Wu, X.R., et al. (2015). Divergent behaviors and underlying mechanisms of cell migration and invasion in non-metastatic T24 and its metastatic derivative T24T bladder cancer cell lines. Oncotarget *6*, 522–536.

32. Sims, J.D., McCready, J., and Jay, D.G. (2011). Extracellular heat shock protein (Hsp) 70 and Hsp90α assist in matrix metalloproteinase-2 activation and breast cancer cell migration and invasion. PLoS ONE *6*, e18848.

33. Pulverer, B.J., Kyriakis, J.M., Avruch, J., Nikolakaki, E., and Woodgett, J.R. (1991). Phosphorylation of c-jun mediated by MAP kinases. Nature *353*, 670–674.

34. Smeal, T., Binetruy, B., Mercola, D.A., Birrer, M., and Karin, M. (1991). Oncogenic and transcriptional cooperation with Ha-Ras requires phosphorylation of c-Jun on serines 63 and 73. Nature *354*, 494–496.

35. Kanayama, H., Yokota, K., Kurokawa, Y., Murakami, Y., Nishitani, M., and Kagawa, S. (1998). Prognostic values of matrix metalloproteinase-2 and tissue inhibitor of metalloproteinase-2 expression in bladder cancer. Cancer *82*, 1359–1366.

36. Moore, C.S., and Crocker, S.J. (2012). An alternate perspective on the roles of TIMPs and MMPs in pathology. Am. J. Pathol. *180*, 12–16.

37. Yan, Y., Liang, H., Li, T., Li, M., Li, R., Qin, X., and Li, S. (2014). The MMP-1, MMP-2, and MMP-9 gene polymorphisms and susceptibility to bladder cancer: a meta-analysis. Tumour Biol. *35*, 3047–3052.

38. Tokuraku, M., Sato, H., Murakami, S., Okada, Y., Watanabe, Y., and Seiki, M. (1995). Activation of the precursor of gelatinase A/72 kDa type IV collagenase/MMP-2 in lung carcinomas correlates with the expression of membrane-type matrix metalloproteinase (MT-MMP) and with lymph node metastasis. Int. J. Cancer *64*, 355–359.

39. Shin, S.S., Park, S.S., Hwang, B., Kim, W.T., Choi, Y.H., Kim, W.J., and Moon, S.K. (2016). MicroRNA-106a suppresses proliferation, migration, and invasion of bladder cancer cells by modulating MAPK signaling, cell cycle regulators, and Ets-1-mediated MMP-2 expression. Oncol. Rep. *36*, 2421–2429.

40. Park, S.L., Cho, T.M., Won, S.Y., Song, J.H., Noh, D.H., Kim, W.J., and Moon, S.K. (2015). MicroRNA-20b inhibits the proliferation, migration and invasion of bladder cancer EJ cells via the targeting of cell cycle regulation and Sp-1-mediated MMP-2 expression. Oncol. Rep. *34*, 1605–1612.

41. Dong, Q., Fu, L., Zhao, Y., Tan, S., and Wang, E. (2017). Derlin-1 overexpression confers poor prognosis in muscle invasive bladder cancer and contributes to chemoresistance and invasion through PI3K/AKT and ERK/MMP signaling. Oncotarget *8*, 17059–17069.

42. Kumar, B., Koul, S., Petersen, J., Khandrika, L., Hwa, J.S., Meacham, R.B., Wilson, S., and Koul, H.K. (2010). p38 mitogen-activated protein kinase-driven MAPKAPK2 regulates invasion of bladder cancer by modulation of MMP-2 and MMP-9 activity. Cancer Res. *70*, 832–841.

43. Dehnavi, E., Soheili, Z.S., Samiei, S., Ataei, Z., and Aryan, H. (2009). The effect of TGF-beta2 on MMP-2 production and activity in highly metastatic human bladder carcinoma cell line 5637. Cancer Invest. *27*, 568–574.

44. Henics, T., Nagy, E., Oh, H.J., Csermely, P., von Gabain, A., and Subjeck, J.R. (1999). Mammalian Hsp70 and Hsp110 proteins bind to RNA motifs involved in mRNA stability. J. Biol. Chem. *274*, 17318–17324.

45. McCubrey, J.A., Steelman, L.S., Bertrand, F.E., Davis, N.M., Sokolosky, M., Abrams, S.L., Montalto, G., D'Assoro, A.B., Libra, M., Nicoletti, F., et al. (2014). GSK-3 as potential target for therapeutic intervention in cancer. Oncotarget *5*, 2881–2911.

46. Yohn, N.L., Bingaman, C.N., DuMont, A.L., and Yoo, L.I. (2011). Phosphatidylinositol 3′-kinase, mTOR, and glycogen synthase kinase-3β mediated regulation of p21 in human urothelial carcinoma cells. BMC Urol. *11*, 19.

47. Chikano, Y., Domoto, T., Furuta, T., Sabit, H., Kitano-Tamura, A., Pyko, I.V., Takino, T., Sai, Y., Hayashi, Y., Sato, H., et al. (2015). Glycogen synthase kinase 3β sustains invasion of glioblastoma via the focal adhesion kinase, Rac1, and c-Jun N-terminal kinase-mediated pathway. Mol. Cancer Ther. *14*, 564–574.

48. Fang, Y., Cao, Z., Hou, Q., Ma, C., Yao, C., Li, J., Wu, X.R., and Huang, C. (2013). Cyclin d1 downregulation contributes to anticancer effect of isorhapontigenin on human bladder cancer cells. Mol. Cancer Ther. *12*, 1492–1503.

49. Guo, W., Liu, J., Jian, J., Li, J., Wan, Y., and Huang, C. (2014). IKK-β/NF-κB p65 mediates p27(Kip1) protein degradation in arsenite response. Biochem. Biophys. Res. Commun. *447*, 563–568.

50. Zhang, D., Li, J., Gao, J., and Huang, C. (2009). c-Jun/AP-1 pathway-mediated cyclin D1 expression participates in low dose arsenite-induced transformation in mouse epidermal JB6 Cl41 cells. Toxicol. Appl. Pharmacol. *235*, 18–24.

51. Song, L., Li, J., Zhang, D., Liu, Z.G., Ye, J., Zhan, Q., Shen, H.M., Whiteman, M., and Huang, C. (2006). IKKbeta programs to turn on the GADD45alpha-MKK4-JNK apoptotic cascade specifically via p50 NF-kappaB in arsenite response. J. Cell Biol. *175*, 607–617.

OMTN, Volume *12*

Supplemental Information

ISO, via Upregulating MiR-137 Transcription, Inhibits GSK3β-HSP70-MMP-2 Axis, Resulting in Attenuating Urothelial Cancer Invasion

Xirui Guo, Haishan Huang, Honglei Jin, Jiheng Xu, Sanjiv Risal, Jingxia Li, Xin Li, Huiying Yan, Xingruo Zeng, Lei Xue, Changyan Chen, and Chuanshu Huang

Supplement Fig. 1

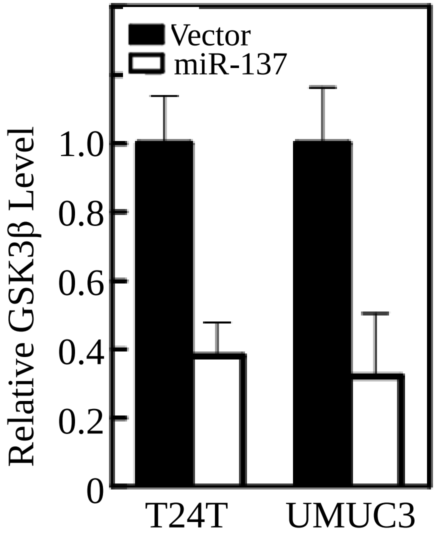

SFig. 1A, Quantitative results obtained from triplicated experiments shown in Fig. 3A.

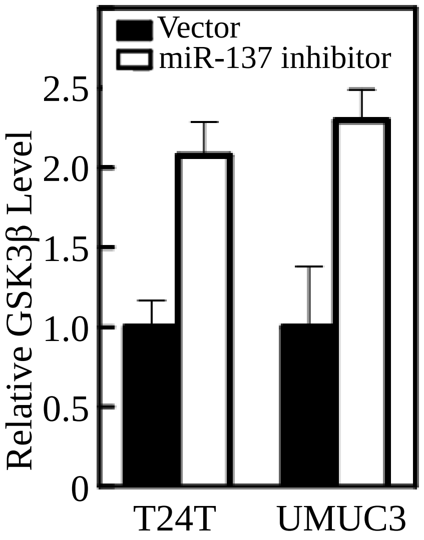

SFig. 1B, Quantitative results obtained from triplicated experiments shown in Fig. 3B.

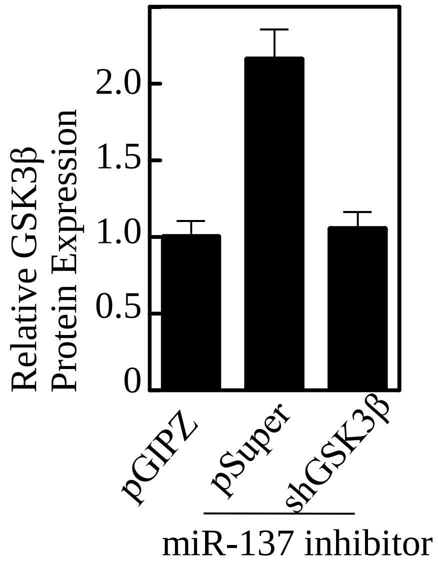

SFig. 1C, Quantitative results obtained from triplicated experiments shown in Fig. 3C.

Supplement Fig. 2

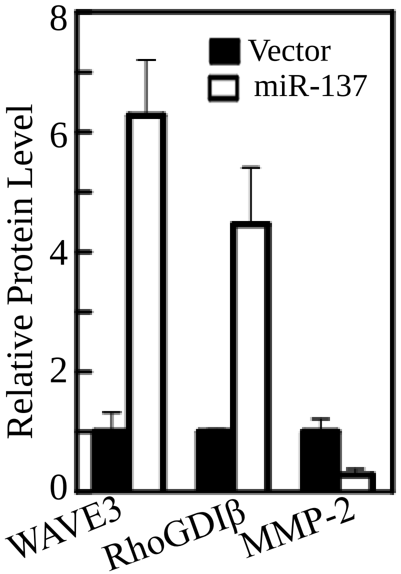

SFig. 2A, Quantitative results obtained from triplicated experiments shown in Fig. 5A.

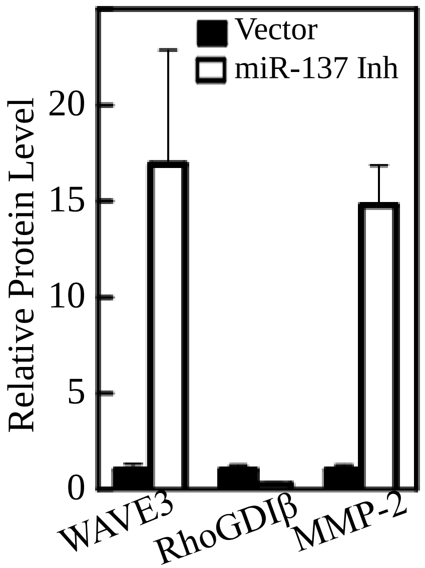

SFig. 2B, Quantitative results obtained from triplicated experiments shown in Fig. 5B.

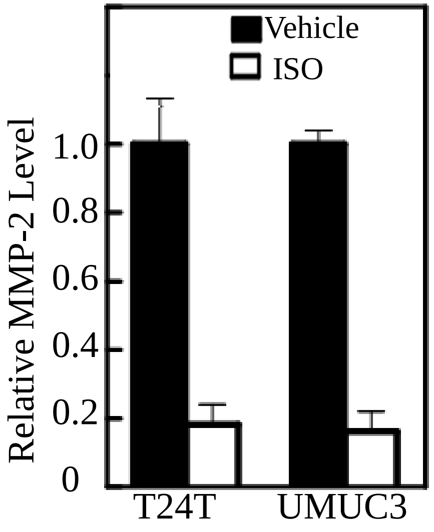

SFig. 2C, Quantitative results obtained from triplicated experiments shown in Fig. 5C.

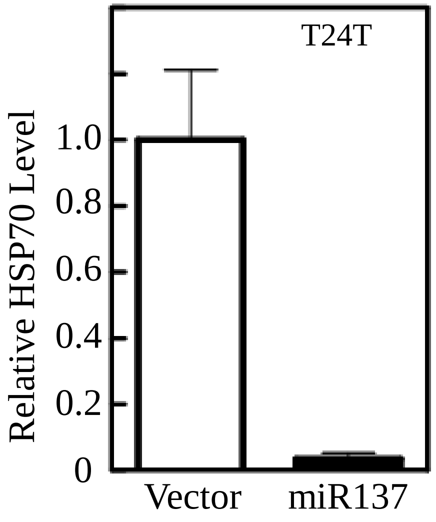

SFig. 2D, Quantitative results obtained from triplicated experiments shown in Fig. 5E.

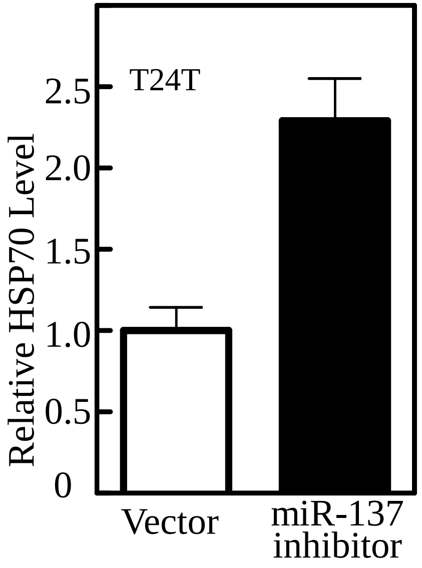

SFig. 2E, Quantitative results obtained from triplicated experiments shown in Fig. 5F.

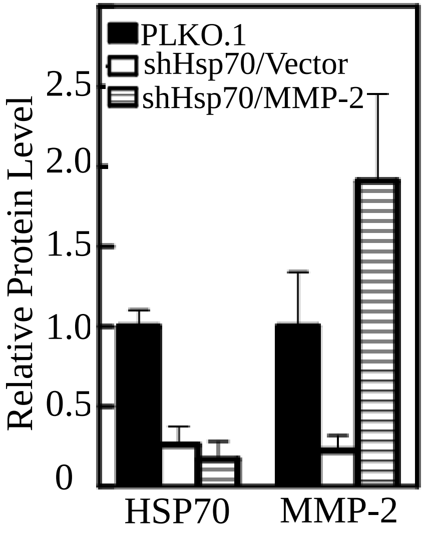

SFig. 2F, Quantitative results obtained from triplicated experiments shown in Fig. 5G.

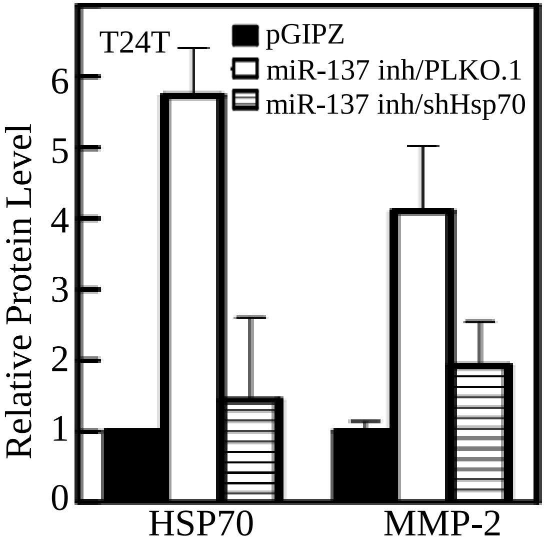

SFig. 2G, Quantitative results obtained from triplicated experiments shown in Fig. 5J.

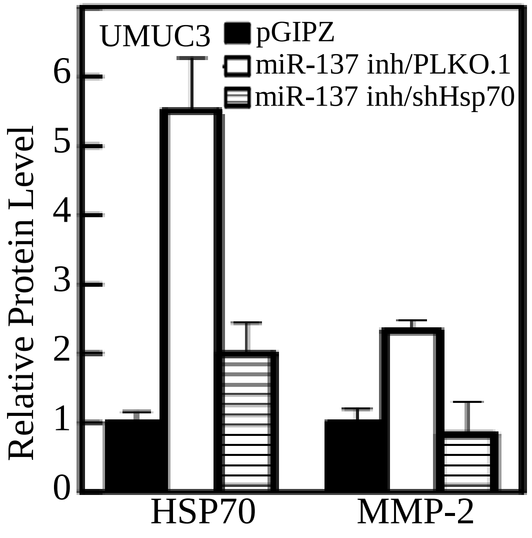

SFig. 2H, Quantitative results obtained from triplicated experiments shown in Fig. 5K.

Supplement Fig. 3

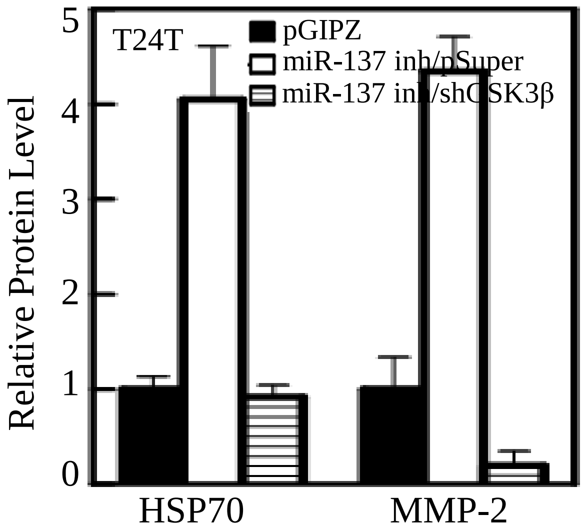

SFig. 3A, Quantitative results obtained from triplicated experiments shown in Fig. 6A.

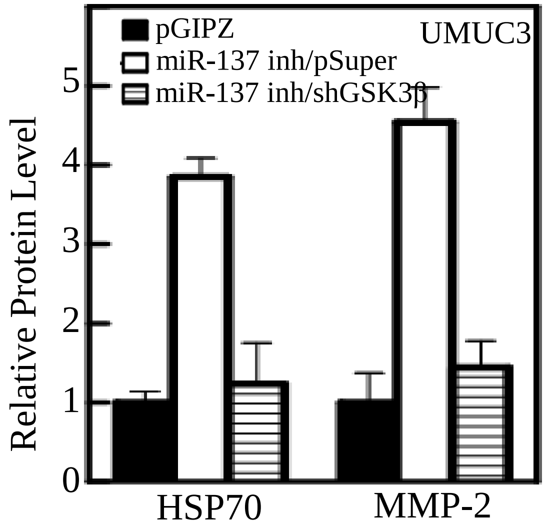

SFig. 3B, Quantitative results obtained from triplicated experiments shown in Fig. 6B.

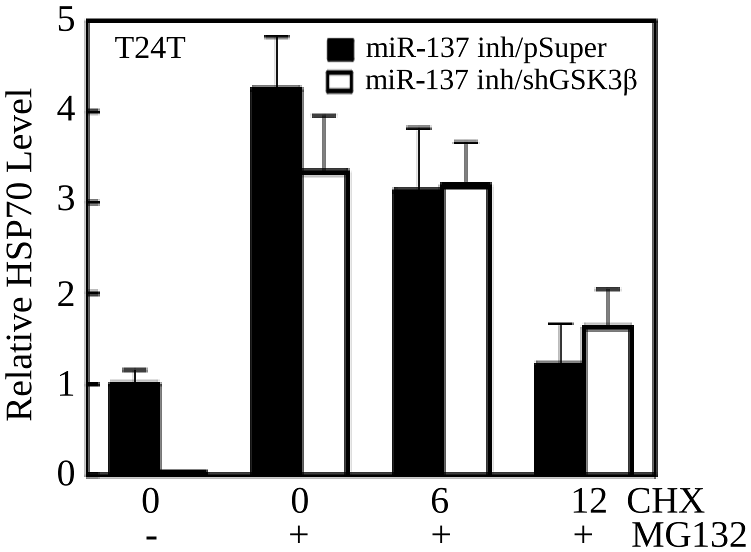

SFig. 3C, Quantitative results obtained from triplicated experiments shown in Fig. 6D.

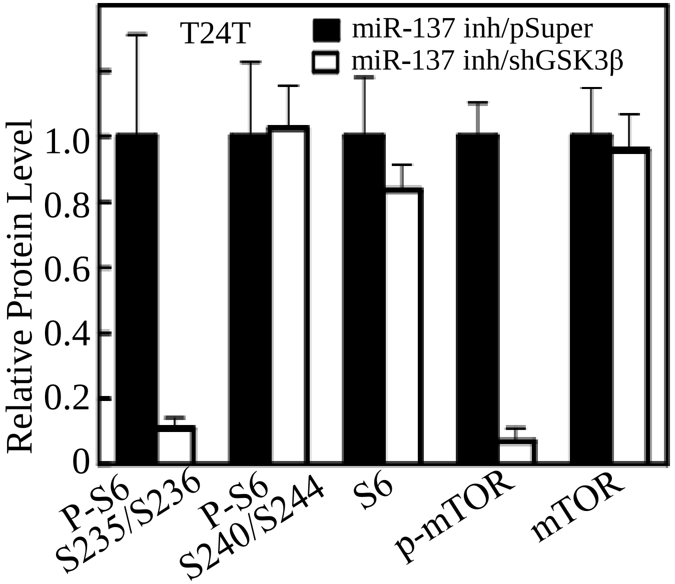

SFig. 3D, Quantitative results obtained from triplicated experiments shown in Fig. 6E.

Supplement Fig. 4

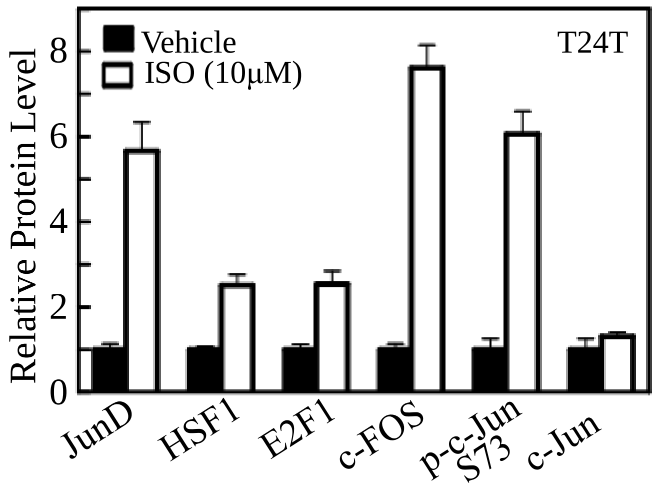

SFig. 4A, Quantitative results obtained from triplicated experiments shown in Fig. 7D.

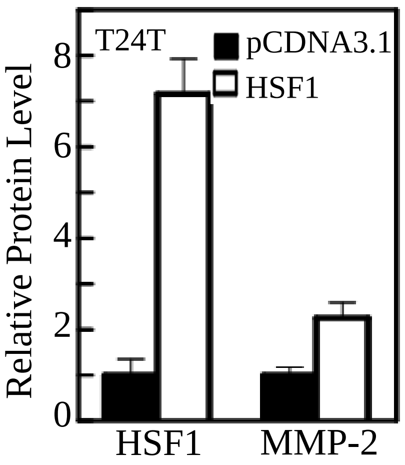

SFig. 4C, Quantitative results obtained from triplicated experiments shown in Fig. 7F.

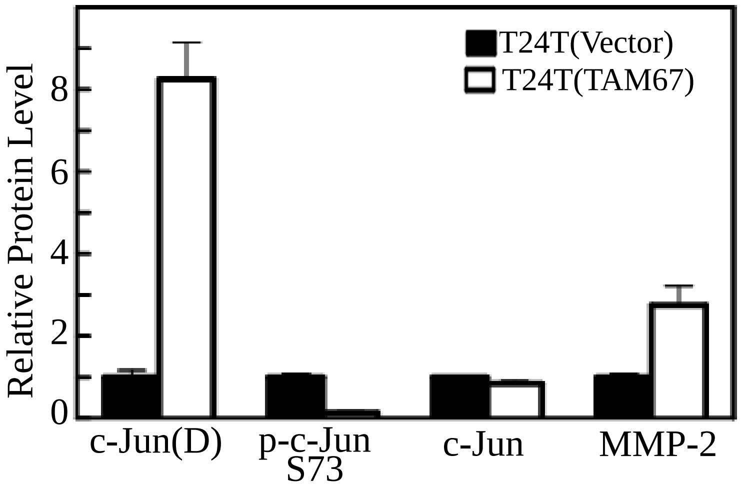

SFig. 4E, Quantitative results obtained from triplicated experiments shown in Fig. 7J.

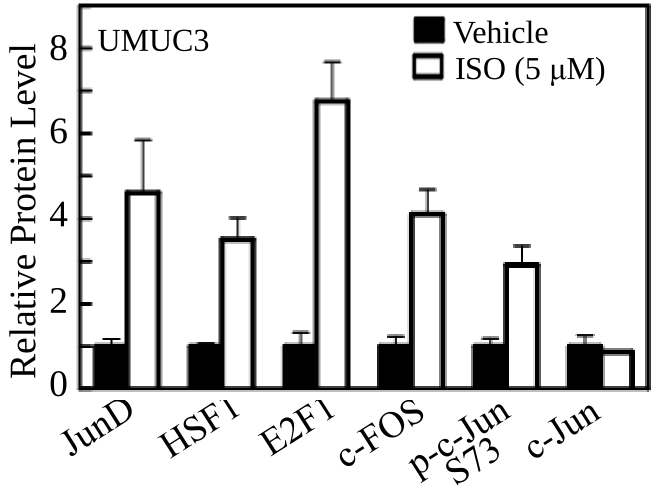

SFig. 4B, Quantitative results obtained from triplicated experiments shown in Fig. 7E.

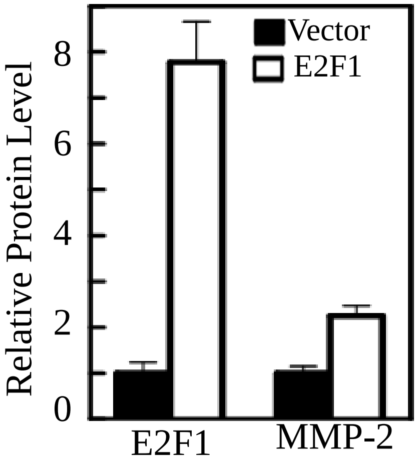

SFig. 4D, Quantitative results obtained from triplicated experiments shown in Fig. 7H.

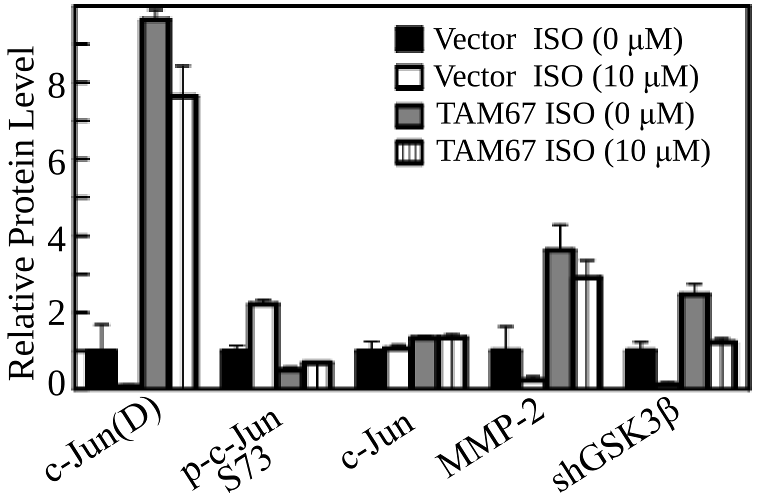

SFig. 4F, Quantitative results obtained from triplicated experiments shown in Fig. 7M.
